# 2+*δ*‐Dimensional Materials via Atomistic Z‐Welding

**DOI:** 10.1002/advs.202202695

**Published:** 2022-09-11

**Authors:** Tumesh Kumar Sahu, Maithilee Motlag, Arkamita Bandyopadhyay, Nishant Kumar, Gary J. Cheng, Prashant Kumar

**Affiliations:** ^1^ Department of Physics Indian Institute of Technology Patna Bihta Campus Bihta Patna Bihar 801106 India; ^2^ Department of Physics Shri Ramdeo Baba College of Engineering and Management Nagpur Maharashtra 440013 India; ^3^ School of Industrial Engineering Purdue University West Lafayette IN 47907 USA; ^4^ Bremen Center for Computational Materials Science Eingang A 28359 Bremen Germany; ^5^ Institute of Technological Sciences Wuhan University Wuhan, Hubei 430074 China; ^6^ Birck Nanotechnology Centre Purdue University West Lafayette IN 47907 USA; ^7^ Global Innovation Centre for Advanced Nanomaterials The University of Newcastle Newcastle 2308 Australia

**Keywords:** 2+ *δ* dimensional, 2D materials, hybridization, hydrothermal, microwave

## Abstract

Pivotal to functional van der Waals stacked flexible electronic/excitonic/spintronic/thermoelectric chips is the synergy amongst constituent layers. However; the current techniques viz. sequential chemical vapor deposition, micromechanical/wet‐chemical transfer are mostly limited due to diffused interfaces, and metallic remnants/bubbles at the interface. Inter‐layer‐coupled 2+*δ*‐dimensional materials, as a new class of materials can be significantly suitable for out‐of‐plane carrier transport and hence prompt response in prospective devices. Here, the discovery of the use of exotic electric field ≈10^6^ V cm^−^^1^ (at microwave hot‐spot) and 2 thermomechanical conditions i.e. pressure ≈1 MPa, T ≈ 200 °C (during solvothermal reaction) to realize 2+*δ*‐dimensional materials is reported. It is found that P_z_—P_z_ chemical bonds form between the component layers, e.g., C—B and C—N in G‐BN, Mo—N and Mo—B in MoS_2_‐BN hybrid systems as revealed by X‐ray photoelectron spectroscopy. New vibrational peaks in Raman spectra (B—C ≈1320 cm^–1^ for the G‐BN system and Mo—B ≈365 cm^–1^ for the MoS_2_‐BN system) are recorded. Tunable mid‐gap formation, along with diodic behavior (knee voltage ≈0.7 V, breakdown voltage ≈1.8 V) in the reduced graphene oxide‐reduced BN oxide (RGO‐RBNO) hybrid system is also observed. Band‐gap tuning in MoS_2_‐BN system is observed. Simulations reveal stacking‐dependent interfacial charge/potential drops, hinting at the feasibility of next‐generation functional devices/sensors.

## Graphical abstract

Novel 2+*δ*‐dimensional materials which are a new class of chemically bonded heterogenous 2D materials are synthesized for the first time.

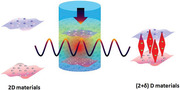

## 1 Introduction

2D materials are uniquely suited for heterolayered devices due to their atomic level flatness, absence of dangling bonds, high surface area, excellent anchoring capabilities, defect‐free/single crystalline nature, standalone quantum mechanical behaviors such as record electron mobility, fractional quantum Hall effect, quantized thermal conductance, etc.^[^ ^1^, ^2^, ^3^, ^4^ ^]^ BN, in particular, provides an atomically smooth surface and epitaxy to graphene and acts as the best substrate for graphene‐based electronic devices, it minimizes electron scattering and enhances the mobility of charge carriers.^[^ ^5^ ^]^ Besides exemplary thermal conductivity, when graphene combines with BN in a stacked manner; one over the other will act as the best thermal packaging tool, in electronic cooling and as a heat spreader for micro‐ or nanoelectromechanical systems and also as laser shield for nanoelectronics circuits.^[^ ^6^, ^7^, ^8^, ^9^ ^]^ Members of dichalcogenides family, e.g., MoS_2_, WS_2_, etc., in bulk possess indirect bandgap (low *E*_g_ in IR range) whereas, semiconductor 2H phase for a few layered systems and co‐existence of direct gap (visible bandgap in green range) semiconductor and metallic 1T phase for monolayers and exhibits moderate mobility (≈300 cm2 V^−1^ s^−1^) in field‐effect transistor (FET) devices.^[^ ^10^, ^11^ ^]^ Electron mobility in atomically thin MoS_2_ has been exploited for fabricating fast electronic chips, which have been employed as prompt ultrasensitive sniffers of poisonous gases and explosives, UV and IR detectors, and THz plasmonic waveguides.^[^ ^12^, ^13^, ^14^ ^]^ Van der Waals stack of different 2D materials has emerged as one of the most sought materials systems due to their tremendous potentials for applications in switching devices, light‐emitting diodes, Hall sensor, resonant tunnel diodes, FETs, high‐speed electro‐optical modulator, photovoltaic devices, interconnects in electronics, plasmonic waveguide modulator, and nonvolatile memory devices.^[^ ^15^, ^16^, ^17^, ^18^, ^19^, ^20^, ^21^, ^22^, ^23^, ^24^, ^25^ ^]^ Besides apparent contrast in the electronic and thermal behaviors of different 2D materials, their lightweight, optical, and electron transparency, the feasibility of atomic‐scale bond folding, and enormous tensile stretchability render them excellent candidate materials to fabricate prospective electronic, photonic, magnetic, and thermal heterolayered metamaterials.^[^ ^26^, ^27^, ^28^, ^29^ ^]^ The opening of tunable bandgap due to Landau level transitions, magneto‐resistance due to Ettingshausen–Nernst effect, new Dirac points due to moiré supper lattice potential, magnetism, unique thermal transport behavior, etc., have already been observed.^[^ ^30^, ^31^, ^32^, ^33^, ^34^ ^]^ Excited‐state energy transfer at the metamaterials' interfaces is of great concern and needs to be adequately addressed.^[^ ^35^ ^]^ Charge transfer between them can also be harvested for fruitful applications.^[^ ^21^, ^36^ ^]^ It should be noted that for efficient interfacial energy and charge transfer, the remarkably strong interlayer coupling between two different 2D materials is desirable.^[^ ^37^, ^38^, ^39^ ^]^ While, existing techniques to fabricate heterolayered devices primarily include in situ sequential crystal growth (which suffers from interdiffusion of atoms at the interface), wet chemical transfer of chemical vapor deposition grown single crystals (which is prone to surface functionality/fluids at the interface), and dry transfer technique (voids/air bubbles/ripples can form in this case) of various layered material by scotch tape or via blind‐blending (uncontrolled orientation and stacking).^[^ ^40^, ^41^, ^42^, ^43^, ^44^, ^45^ ^]^ Pivotal applications of these novel cutting edge advanced materials in futuristic ultra‐sensitive functional devices and sensors are their synergistic integrated response, e.g., out‐of‐plane carrier (electronic, spintronic excitonic, photonic, or thermal) transport. Such integrated response would be dependent on how the component layers are coupled among themselves. Interlayer distance, stacking sequence of individual layers, the relative angle between atomic sheets, and localized strain which would pull atoms out‐of‐plane are various factors working in sync with each other determining the overall crystallographic structure and hence electronic/optical/optoelectronic or thermal/thermoelectric behaviors of the artificial crystals. Existing synthesis methods and fabrication techniques are suffering from dirty interfaces (due to defects/functionalities/fluids/voids at the interface) which results in poor interlayer coupling, which has limited practical realization of pairing quantum states in these quantum systems, demonstration of their exotic behavior and their exploitation in potential flexible devices aimed at ultrafast sensing, excitonics, in renewable energy generation and storage. In order to improve the poor quality of the interface, to fix the serious concern of misorientations of individual 2D crystals which potentially can impede carrier transport, it would be a great milestone to achieve if the dream of having 2D crystals chemically bound out‐of‐plane can practically be realized. Such out‐of‐plane binding will help in fast‐tracking carriers, and it would potentially bring in synergy among them to act as a single unit and to provide an integrated response to stimuli. In fact, the scientific community has long been waiting for this discovery.

Thinking beyond the apparent, realizing chemical binding between individual component 2D materials would help attain a unique new class of materials (2D materials interlayer‐coupled, i.e., having overall effective dimensionality exceeding 2, i.e., 2+*δ*‐dimensional materials) which have earlier been deemed as metamaterials with a unique identity, features, and functionality. An integrated collective response is expected from such advanced materials. 2D materials being quantum mechanical in nature, 2D materials hybrids (or, 2+*δ*‐dimensional materials) are supposed to have quantum mechanical energy states with access to new hybrid states (inaccessible otherwise in individual atomic sheets). Orbital hybridizations between atomic sheets were experimentally realized by a) volume confinement and b) under exotic conditioning, namely, hydrothermal (pressure and temperature) and microwave (electric field and temperature) techniques. While hydrothermal treatment involves moderate temperature and high‐pressure processing of materials, microwave irradiation involves high local temperature and intense electric field treatment. Obtained results were compared with that for direct heating, which is routine these days. Apart from high‐resolution transmission electron microscopy (HRTEM), UV‐vis, photoluminescence, Raman, X‐ray photoelectron (XPS), and Fourier transform infrared (FTIR) spectroscopies were employed to assess interlayer coupling and its impact on functionalities. Electrical testing of samples with and without light, and also with and without strain was carried out. To ascertain carrier type and to evaluate carrier concentration, Hall effect was employed. Moreover, to pinpointedly understand interlayer coupling, the electric field effect on Raman spectroscopy was also carried out. To verify our experimental finding of interlayer coupling, molecular dynamics (MD) simulations were carried out with 2D materials, equilibrium distances were obtained under different processing conditions, and with the emerging atomic configurations, density functional theory (DFT) band structure calculation was carried out to attain density of states and charge density difference profiles. The products obtained by hydrothermal as well as microwave processing were employed for strain sensing, gas sensing, polyvinylidene fluoride‐based thermoplastics, and controlled surface wettability behaviors.

## 2 Results and Discussion

**Figure 1:**
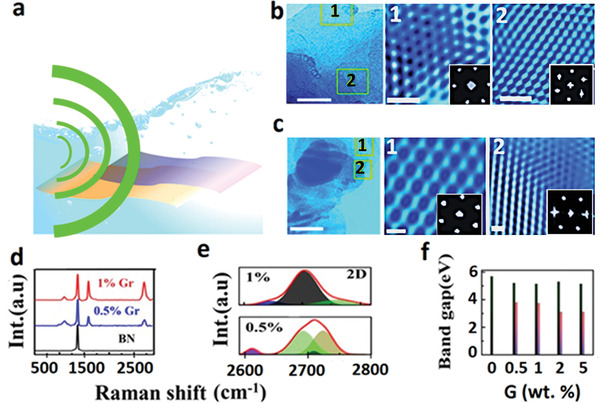
a) Schematic diagram of sonochemical hybridization process. b) TEM image for 1 wt% of Gr‐BN and its HRTEM images in overlap areas marked in TEM as 1 and 2, insets corresponding to their FFT pattern. c) TEM image for 0.5 wt% Gr‐BN system overlapped areas confirmed by elemental mapping marked as 1 and 2 and their HRTEM images, in inset corresponding FFT patterns f) bandgap histogram for hybrid systems with different wt% (0 to 5 wt%) of Gr in BN obtained from UV‐visible measurements and d) Raman spectra for hybrids (0.5 to 1) wt% of Gr in BN and e) deconvolution of 2D peaks for hybridized (0.5 and 1 wt%) samples.

Bringing two distinct 2D materials with contrast in physical/chemical behavior has been intriguing as it promises an excellent platform to maneuver material properties. Instead of blind blending, dilute solutions having graphene and boron nitride have been reported to yield mid‐gaps.^[^ ^44^ ^]^ To have a glimpse of vertical hybridization between Gr and BN systems, graphene‐BN blends were sonochemically synthesized up to 5 wt% of graphene with BN as a matrix in the present research (see Figure S1 in the Supporting Information and schematics in **Figure** **1**a). Interlayer distance and angle apart from sequence of material layers and choice of materials are responsible for eventually determining the atomic configurations in resulting blends (see Figure 1b and their overlap areas in 1 and 2) for blend having 0.5 wt% graphene and (see Figure 1c and their overlap areas in 1 and 2) for blend having 1 wt% of graphene. In fact, we obtained distinguishable atomic ordering in different locations (hexagonal atomic ordering (sixfold in Figure 1a1,b1) and atomic ordering having mixed folds (sixfold and twofold together in Figure 1a2,b2)). Atomic profiles taken on these HRTEM images (see Figure S2, Supporting Information) with distinct atomic structures reveal that average interatomic distance across the atomic arrangement was calculated to be 3.0 Å and along the atomic line found to be 2.3 Å with an angle between the symmetry lines to be ≈47 ° for 0.5 wt%, similarly for 1 wt % atomic distance across the atomic arrangement was calculated at 3.0 Å, and along the atomic line, it was 3.1 Å with an angle between the symmetry lines to be 58°. Fast Fourier transform (FFT) acquired for the selected area further supports the details emerging from HRTEM atomic imaging, especially relative distances and angles. The bandgap of BN theoretically is 6.1 eV, however experimentally, it is ≈5.6 eV due to partial stacking of BN layers during synthesis. Fragmentation of BN sheets which exposes edges gives rise to other peaks, mild though. Tauc plots obtained from UV‐vis spectroscopy of dilute solution having graphene‐BN blend reveal that mid‐gaps reduce when blending % is enhanced (see Figure 1f). Apart from routine D and G peaks at ≈1353 and ≈1583 cm^−1^ in both the blends, we observed E_1g_ vibration mode due to BN present in both the cases, peak shifts in G (3 cm^−1^ for 0.5% and 2.7 cm^−1^ for 1% graphene loading) and BN E_1g_ vibration mode ≈1360 cm^−1^ shifted (4.5 cm^−1^ for 0.5% and 4 cm^−1^ for 1%) similarly 2D peaks ≈2690 cm^−1^ for graphene shifted in hybrid material (13.7 cm^−1^ for 0.5% and 1.4 cm^−1^ for 1% loading) further validate hybridization. The 2D peak is sensitive to layer stacking, and therefore, it was deconvoluted for both a maximum of 0.5 and 1 wt% graphene in G‐BN blends. Apparently, 1% blend exhibits the signature of enhanced stacking. Δ*ω* observed for deconvoluted 2D peaks is more for 0.5% concerning 1% sample. The emergence of new Raman modes (vertical vibrations) at ≈1100 and ≈1320 cm^−1^ (B‐C) vibration in such blends is an interesting result confirming interlayer coupling. In fact, it establishes atomic bindings between C and B (of BN molecule) and thus hinting at the possible cooperative or integrated response by the hybrid material system to the external trigger (if any); however, bond formation needs to be explored further by other techniques though (see Figure 1d,e).

**Figure 2:**
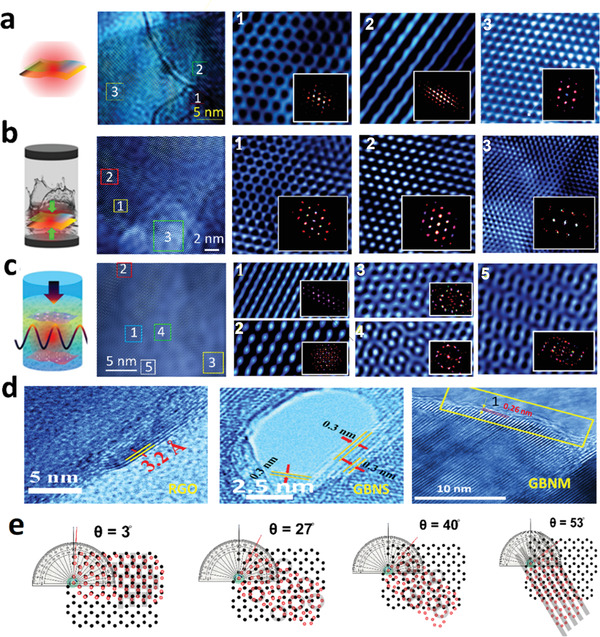
Schematic diagram of hybridization approaches, HRTEM image, and zoomed‐in images for a) GBNH, b) GBNS, c) GBNM. Hexagonal symmetry is found in individual atomic sheets of G and BN. In hybrids, new symmetries evolve and mixed symmetries are found. To resolve local structural features, zoomed‐in images are shown. Interesting structural patterns of atoms are registered. Microwave (*P*+*T*+*E*) technique exhibits intense hybridization followed by the solvothermal (*P*+*T*) technique and heating (*T*) had the least effect. d) Schematic showing evolution of moire patterns at various angles between G and BN. Stripes, linear chains, circular (single) array, tricircle joining each other and various other features are generated at different angles.

Hybridization between graphene and BN was explored for chemically synthesized RGO and RBNO too. TEM was employed extensively to have a glimpse of how exotic reaction conditions such as solvothermal or microwave treatment impact hybridization and interlayer coupling of resulting product materials. When synthesized, graphene oxide (GO) is mixed with BN oxide (BNO) with different wt% as shown in‐camera image (see Figure S7, Supporting Information) in dimethylformamide (DMF) solvent and sonicated for 3 h using ultrasonication for proper mixing. Mixed solutions are heated at 300 °C on the hot plate until all solvent gets evaporated and facilitate partial heat‐mediated (*T* only) hybridization (GBNH sample) as shown in **Figure** **2**a. While graphene or BN individual layers exhibit sixfold symmetries and show hexagonal FFT patterns, GBNH exhibits twofold and mixed‐fold (2+4+6) symmetries (see Figure 2a). The obtained powdered mixture was then treated separately in DMF solvent in a solvothermal autoclave (*P*+*T*) giving sample GBNS (see Figure 2b) and microwave oven (*P*+*T*+*E*) giving sample GBNM (see Figure 2c). The choice of DMF solvent has been made as it is a reducing solvent. GBNS exhibits enhanced hybridization as compared to GBNH (see Figure 2b). Interestingly, GBNM exhibits several new patterns in HRTEM zoomed‐in versions and also is displayed in very interesting FFT patterns. The existence of voids, very bright spots, ring‐like and triplet ring‐like features are obtained in GBNM (see Figure 2c). These interesting local atomic‐scale structural features in HRTEM zoomed‐in images hint at efficient hybridization.

To confirm the effect of hybridization on the interlayer distance, we have minutely analyzed the interlayer distance in GBNS and GBNM samples and compared them with the RGO sample. When RGO was analyzed with TEM measurement, it exhibited highly transparent monolayer sheets as shown in Figure 2d. The interlayer distance was ≈3.2 Å as measured in the HRTEM image. In contrast to RGO, GBNS and GBNM exhibited interlayer distances of ≈3.0 and ≈2.6 Å (see Figure 2d). The change in carbon—carbon bond lengths implies that the effect of hybridization is not solely restricted to interlayer distances but in‐plane atomic reconstructions have also been observed at the hybridization sites. In fact, moire patterns in 2D–2D hetero layers due to stacking at particular angles are well known and have made news recently due to unusual electron coupling which gives superconductivity, especially at magic angles (≈1.12°). Even without moire patterns, the stacking sequence can be AA or AB stacking. Moire patterns generation in general can be extended to even higher angles (see Figure 2e). As one can see various patterns grow at various angles. Even translation can be coupled with the twist and new features can evolve. While the moirés pattern signifies the strong interlayer coupling accomplished via strong hybridization, reconstruction and new structural evolution hint at intense orbital hybridization. The possibility of out‐of‐plane bond formation is therefore imminent and needs to be diagnosed further. It should be noted that such bond formation is field catalyzed and thermodynamically driven. Apart from revealing proofs such huge peak shifts/new peaks in X‐ray diffraction (XRD) due to new out‐of‐plane bond formation (Figure S3, Supporting Information), FTIR peak shifts (Figure S4, Supporting Information), and Raman peak shifts Figure (Figure S5, Supporting Information), XPS characterization (details in Figure S15, Supporting Information) profoundly reveals vertical C—N bond formation >25% for GBNS and ≈50% for GBNM. Optical as well as electronic band gap and carrier density has been measured on G‐BN hybrid samples obtained by various approaches (see Figures S19 and S21, Supporting Information). Mid‐gap formation down to ≈3.2 eV is observed.

Solvothermal synthesis has been employed for various synthetic pursuits such as for pure graphene quantum dots, gCN, graphitic BCN, etc. It should be noted that C—N (305 kJ mol^−1^) and C=N (615 kJ mol^−1^) bonds form in the solvothermal synthesis of crystalline carbon nitride. Compared to furnace heating (*T* only) where crystallization is limited by temperature only and heating below crystallization temperature falls short of crystallization, solvothermal synthesis involves both temperature and pressure (*T*+*P*). In a typical solvothermal reaction, *T* used is ≈200 °C and the container filled with boiling solvents (low BP) for typical 24 h creates a pressure of ≈1 MPa. Pressure reduces the enthalpy of the formation of these bonds. In microwave synthesis, other than *T* and *P*, a local electric field >10^6^ V cm^−1^ is available. It should be noted that due to high microwave absorption by the G+BN mixture, the local temperature can exceed 2000 °C. *T*+*P*+*E* work simultaneously. While solvothermal synthesis is pressure catalyzed, microwave synthesis is primarily field catalyzed.

Atomic distance in the hybrid system was obtained from HRTEM by measuring atomic distance along the atomic line and across the atomic line. It turns out that the observed average atomic distance along the atomic line was 3.3 Å in contrast to 3.5 Å across the atomic line (see details in Figure S8d, Supporting Information). Similarly, for the GBNS hybridized system, due to pressure and chemical energy inside the vessel at fixed temperature ≈200 °C for 4 h, it is expected that RGO and RBNO will further come close to each other and hence hybridization will further get enhanced due to strong interlayer coupling. The presence of RGO and RBNO was confirmed by elemental mapping (see Figure S8b, Supporting Information). The average atomic distance obtained from HRTEM in solvothermal hybridized RGO‐RBNO sample across the atomic line was 3.2 Å, whereas, along the atomic line, it exhibited 2.2 Å (see Figure S8e, Supporting Information). The atomic distance along the atomic line decreases ≈33% significantly compared to the heat‐mediated hybridized (RBNO‐RGO) sample, suggesting the strong influence of foreign atoms and bond formation with the host atomic sheets. When the mixed solution of (RGO‐RBNO) in DMF solvent brisked to microwave at 750 W for 5 min in pulse manner at intervals of 1 min, additional energies are supplied in the form of an electric field compared to chemical and pressure energies as in the solvothermal method. Such pressure and electric field‐induced hybridizations can significantly tune the dielectric properties of hybrid materials and have different dielectric constants concerning their parent materials. When microwave was exposed to RGO‐RBNO system, BN exhibits slightly polar behavior (charge misbalance between B and N); ionic polarization will be a more important factor for hybridization of BN with RGO. The coupling between ionic polarization and electric field exhibited more responsiveness toward the microwave hybridization process. Electric permittivity along the electric field line and perpendicular to the field line depends upon the number of layers in (RBNO and RGO), for monolayer BN it is (*ɛ*ǁ = 5.37) and (*ɛ*□ = 1.82) and for graphene (*ɛ*ǁ = 14) and (*ɛ*□ = 3.0) at 2.45 GHz frequency. High dielectric value across the electric field line made RGO a strong microwave absorber compared to RBNO; thus, the thermal expansion of the RGO lattice will occur. Simultaneously, due to quantum confinement, BN will allow the field line to pass more toward RGO. Hence, RGO will get more active sites for hybridization due to the reduction of functional groups from its basal planes. Besides, the synergistic effect of electric field coupling with the ionic polarization of RBNO makes it even more responsive to hybridization with RGO as the cumulative effect of (heating, pressure, and electric field) inside the reaction vessel interlayer coupling is expected to be the strongest.

Electrostatic interaction between RGO‐RBNO gets influenced by an external electric field (106 V cm^−1^), and hybridization became more prominent as a result, partially 3D behavior with highly strained sheets appeared in HRTEM. When TEM measurement was performed in the hybridized sample, highly transparent sheets were observed with multiple overlap areas. These overlap areas were 50–80 nm in length; elemental mapping confirmed the presence of RBNO and RGO sheets (see Figure S8c, Supporting Information). The average atomic distance measured from obtained HRTEM along with the atomic arrangement array was 2.3 Å. In contrast, across the atomic line, it was 2.17 Å, which is 34.2% less than the observed atomic distance in the case of GBNH (see Figure S8f, Supporting Information). The decrement in the atomic distance for microwave‐hybridized samples is possibly due to strong bond formation between RBNO and RGO sheets as an outcome partially 3D distortion with highly strain sheets would form.

**Figure 3:**
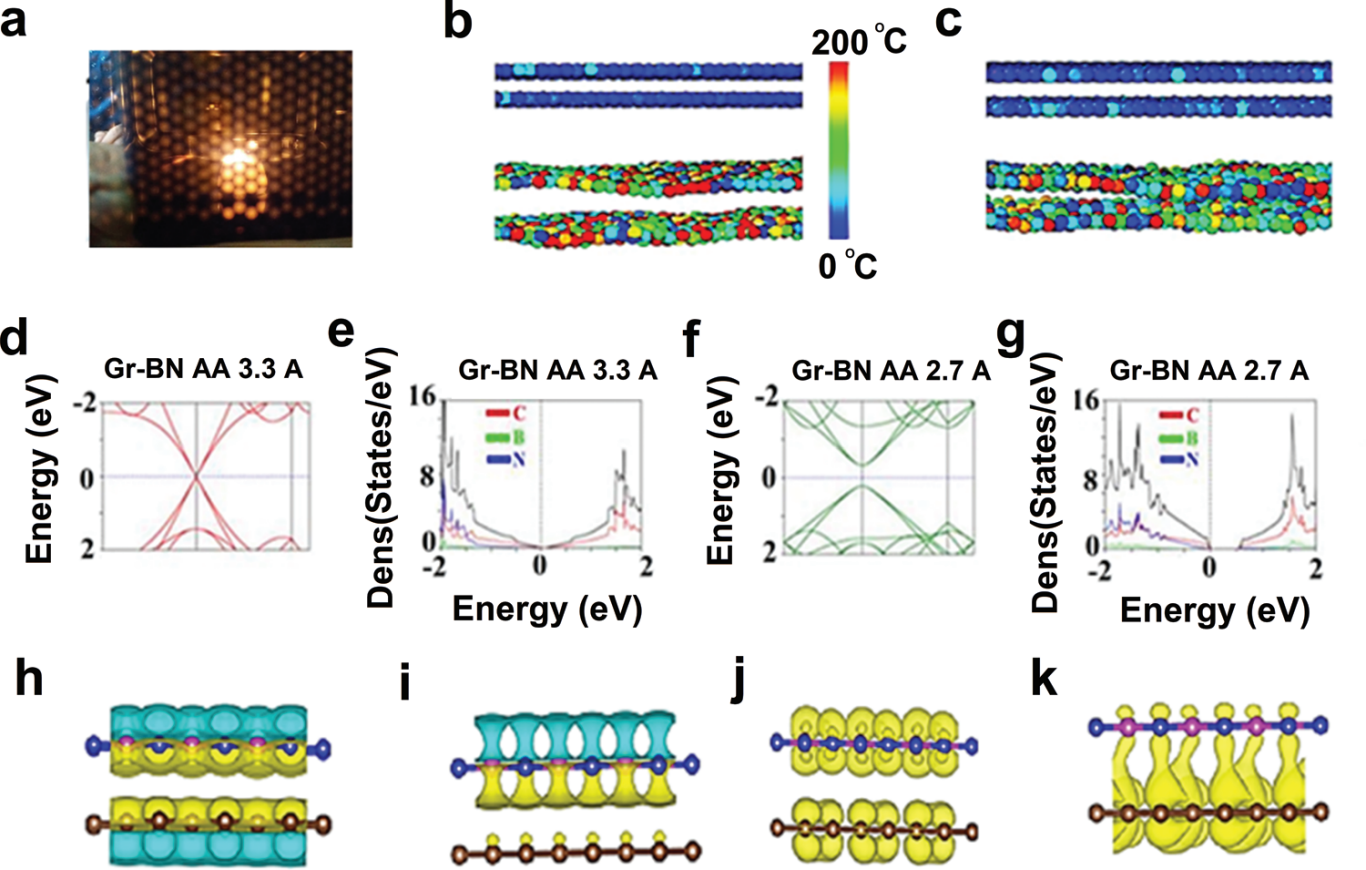
a) Camera photo of the plasma generated during microwave processing. b) Molecular dynamics simulation for Gr‐BN solvothermally hybridized sample at equilibrium condition with average interlayer distance 3.3 Å and c) when hybridized, their interlayer distance locally decreased to 2.7 Å (color index exhibits localized temperature of the atoms). d) DFT band structure calculation for reduced Gr‐BN system considering AA stacking with interlayer distance 3.3 Å and e) DOS calculation for the Gr‐BN system with an insignificant band gap opening. f) DFT band structure calculation for AA stacking for Gr‐BN system with interlayer distance 2.7 Å in E‐k diagram exhibits parabolic dispersion with band gap opening and g) DOS calculation when the interlayer distance was 2.7 Å with AA stacking for Gr‐BN system which exhibits bandgap of 0.5 eV. h,i) Top and side views of charge density difference profile for Gr‐BN system in AA sequence with equilibrium distance 3.3 Å (electron transferred from P_z_ orbitals of Gr to P_z_ orbitals of BN). j,k) Charge density difference profile for reduced Gr‐BN system in AA stacking sequence with reduced distance 2.7 Å electron transfer from P_z_ orbitals of RGO to P_z_ orbitals of RBNO with physical bond formation due to strong interlayer coupling.

Exploring the effect of dynamic conditions in solvothermal and microwave and their sequel on hybridization, we performed MD simulation and DFT band structure calculation to figure out the possible bandgap opening. When MD simulation was performed for a microwave hybridized sample for Gr and BN sheets, it was considered adequate temperature and pressure inside the vessel. Camera photo of the plasma (see **Figure** **3**a) generated during the microwave exposure provides visible evidence of exotic conditions of *T*+*P*+*E*. In equilibrium, the interaction between Gr and BN was minimal with the interlayer distance of 3.3 Å (see Figure 3b). After hybridization by solvothermal method, bond formation occurred due to interlayer coupling, which results in local distortion in atomic arrangements (see Figure 3c) (crumpling due to strain and out‐of‐plane bond formation); because of hybridization, localized charge transfer takes place, and hence the localized distances decrease to 2.7 Å. DFT band structure and charge density difference profile were obtained by considering AA and AB stacking of Gr and BN sheets (see details in Figure S22, Supporting Information). In AA stacking (Gr‐BN) at equilibrium distance, ≈3.3 Å sharp conic linear dispersion was observed in E‐K with the valance band touching the conduction band at Γ point, the same behavior was displayed in the density of state as well, where the conduction band and valance band touching each other at Γ point (see Figure 3d,e). When interlayer distance decreased from 3.3 to 2.7 Å (see Figure 3f,g), significant distortion from sharp conic linear dispersion behavior to parabolic dispersion was observed with bandgap opening at the Γ point. The density state also suggests the bandgap opening of 0.5 eV, although DFT does not consider dynamic conditions (pressure and temperature); it is expected that it will underestimate the bandgap's value. Similarly, DFT band structure calculation was performed by changing the distance (4.5–3 Å) (see Figure S17a–d, Supporting Information) for AA stacking sequences, and it was found that as distance changed from 4.5 to 3 Å, broadening with slight parabolic nature was observed in E‐k curve for conduction band and valance band which further suggests the strong influence of foreign atoms (B and N) in the vicinity of Gr sheets. DFT and density of state (DOS) calculation for AA stacking were compared with the AB stacking sequence when the distance was kept at a point of 2.7 Å. In AB, stacking dispersion was slight parabolic in nature with bandgap opening (≈0.2 eV) in contrast to AA stacking where band gap was found to be 0.5 eV due to strong hybridization, which was also supported by DOS measurement (see details in Figure S18a–d, Supporting Information). The charge density difference profile gives further insight into the hybridization process at 2.7 Å in the Gr‐BN hybrid system where AA stacking was present at different E‐K points (see details in Figure S22, Supporting Information). When Gr and BN were brought close to each other at an equilibrium distance of ≈3.3 Å, electrons transferred from P_z_ orbitals of Gr to P_z_ orbitals of BN as shown in Figure 3 (h) top view and i) side view); here, we have not observed any bond formation (just interacting system). When a similar profile was generated for a lower distance of 2.7 Å, charge transfer became more prominent along with the P_z_—P_z_ chemical bond formation between BN and Gr layers as witnessed in Figure 3 (j) top view and k) side view), such bond formation is due to strong interlayer coupling.

**Figure 4:**
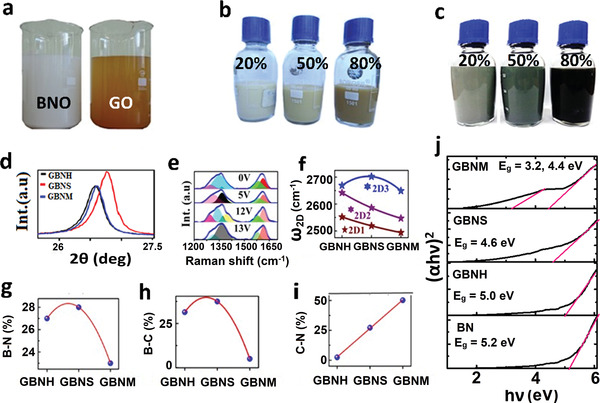
a) Camera images of BNO and GO as synthesized by modified Hummer's method. b) Typical GBNH samples with 20%, 50%, and 80% of RGO in RBNO. c) Typical GBNM samples with 20%, 50%, and 80% of RGO in RBNO. d) XRD, e) electric field‐dependent modulation of Raman spectra of 50:50 GBNM hybrid. f) 2D sub‐peaks (deconvoluted) positions, g) B‐N %, h) B‐C %, and i) C‐N % attained by XPS analysis in GBNH, GBNS, and GBNM samples. j) Tauc plot for BN, GBNH, GBNS, and GBNM samples.

While GO and BNO (synthesized by modified Hummer's method) have the orange and white visual appearance (see **Figure** **4**a and Figure S7, Supporting Information), their typical GBNH and GBNM hybrid samples at 20%, 50%, and 80% appear in different shades (see Figure 4b,c and Figure S7, Supporting Information). We observed a new XRD peak at 2*θ* ≈54.9° due to B_4_C‐like structure (see Figure 4d and details in Figure S3 and Section S1, Supporting Information), Raman peak shifts for D and G and 2D peaks (see Figure 4e,f and Figure S5, Supporting Information) further demonstrate the effect of hybridization. It should be noted that we used an external electric field to probe the coupling and at higher voltage, it exhibits 1320 and 1328 cm^–1^ new Raman bands corresponding to out‐of‐plane C—N and B—C bonds. In FTIR measurement, we observed a new mode (C‐N), which appeared at ≈1727.6 cm^–1^ (see Supporting Information for details). XPS measurements further validated hybridization in GBNH, GBNS, and GBNM (see details in Figure S15 and Section S4, Supporting Information). In brief, while exotic thermodynamic conditions brought by *P*+*T* or *P*+*T*+*E* reduce B—N bond %, the C—N bond % significantly enhanced (see Figure 4g–i). As an effect, the physical properties of the hybrids can be tunably changed at will. For example, while BN monolayer has a theoretical bandgap of 6.1 eV, RBNO exhibits 5.2 eV (possibly due to the presence of a few layers as well); GBNS and GBNM exhibit mid‐band gaps ≈4.4 and 3.2 eV, respectively (see Figure 4j and Figure S19, Supporting Information).

In order to generalize the concept of hybridization, we went ahead to synthesize various other sets of samples such as BN‐MoS_2_, MoS_2_‐WS_2,_ etc. Detailed microscopic and spectroscopic characterizations have been carried out and presented extensively in the Supporting Information. For example, insulator RBNO and semiconductor MoS_2_ system hybridized (RBNO‐MoS_2_) by three different techniques, namely, heating (BNMH), solvothermal (BNMS), and microwave (BNMM) were diagnosed with XPS measurement (see details in Section S4 and Figure S16, Supporting Information). In brief, upon hybridization, it is expected that various bond formations (Mo—B and Mo—N) will occur. Mo—N bond % was calculated from N 1s and Mo 3d spectrum and was found at 63.5% for BNMH, 71% for BNMS, and 62.5% for BNMM samples. Likewise, Mo—B bond % was estimated after the deconvolution of Mo 3d and B 1s spectrum, 23.4% for BNMH, 20.3% for BNMS, and 23.4% for BNMM hybridized samples. Hybridization and their bond formation are also validated by Raman measurement. It exhibited a distinct Raman active vibration mode 365 cm^−1^, which corresponds to the Mo—B bond. The Mo—B signature was also demonstrated by XRD measurement, where different peaks at ≈39.4° and 44.0° were commensurate with the MoN‐like structure. Similarly, peaks at ≈34.4° and 60.1° are analogous to XRD peaks from Mo_2_B‐like structure (see details in Section S1 and Figure S3c,d, Supporting Information). Likewise, we have hybridized MoS_2_ and WS_2_ (see camera image in Figure S12 in the Supporting Information for details about the synthesis process in bulk scale using three techniques). XRD obtained from the MoS_2_‐WS_2_ hybrid system was thoroughly examined for three different techniques. Interestingly, we observed that d‐spacing decreased (see details about XRD measurement in Section S1 and Figure S3e,f, Supporting Information). Similarly, in Raman measurement, in‐plane and out‐of‐plane vibrations are sensitive to any change in the surrounding medium. We observed a shift in E_2g_ and A_1g_ vibration modes of MoS_2_ and found a significant shift in ∆*ω* 27.24 cm^−1^ for heat‐mediated (MWH) hybridized and 25.8 cm^−1^ for solvothermal hybridized (MWS) samples (see more details in Section 2 and Figure S5i–l, Supporting Information).

The hybridized sample will be fascinating to observe by TEM measurements as different atomic arrangements are expected for different lattice orientations. We performed TEM measurements on hybridized systems (see details about TEM measurement in Section S3 and Figures S12 and S13, Supporting Information). Semiconducting materials are of the utmost importance due to the device aspect. Tuning their hybridization will provide a new avenue for designing desirable electrical bandgap material. In this context, we carried out the *R* versus *T* measurement for the MoS_2_‐RBNO hybrid to figure out the electrical bandgap and found that we have achieved the bandgap tunability from 0.3 to 0.5 eV (see details in Figure S20, Supporting Information). When DFT band structure calculation was performed for the BN‐MoS_2_ hybrid considering MoS_2_ as a semiconducting monolayer with 1.98 eV, when the interlayer distance was reduced from 3.3 to 2.7 Å, it exhibited a bandgap of 2.7 and 2.9 eV, suggesting the strong hybridization due to interlayer coupling and bond formation (see details in Figure S23, Supporting Information). Thus, 0.3–0.5 eV bandgap experimentally measured in our study hints at the formation of 1T metallic phase of MoS_2_ due to the processing under exotic conditions of pressure–temperature or electric field pressure. BN monolayer is an insulating material with a large bandgap (≈6.1 eV), whereas graphene is semimetal (≈0 eV). Hybridization of RBNO and RGO by various methods (heating, solvothermal, and microwave) will lead to strong interlayer coupling.

**Figure 5:**
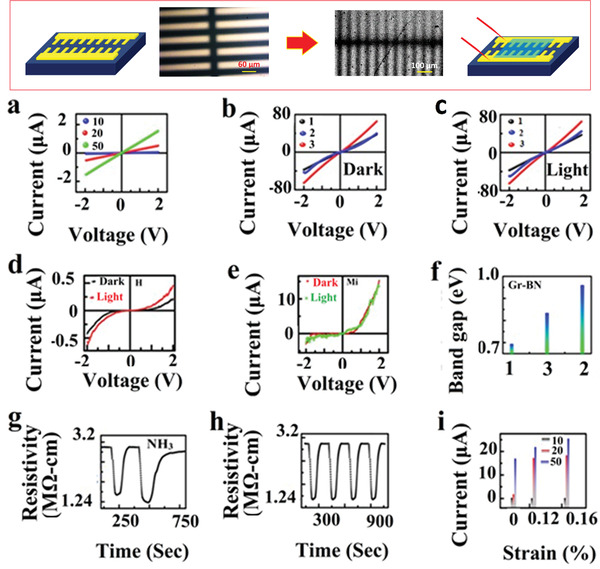
a) Two probe *I*–*V* measurements of hybridized RGO‐RBNO wt% at (10, 20, and 50) samples spin‐coated on PET substrate, conductivity increased with increasing % of RGO in BN from 0.1 to 1.8 µA. b,c) In‐plane *I*–*V* measurements with and without a light source for (GBNH, GBNS, GBNM) hybridized samples, GBNM hybrid exhibited the highest current values 65 µA with semiconducting nature compared to GBNS (40 µA) and GBNH (35 µA), and the highest photocurrent response was recorded for GBNS ≈46 µA. d,e) *I*–*V* measurements out‐of‐plane for GBNM and GBNS exhibited tunneling currents of 15 and 0.3 µA in the dark; upon exposure to blue light, it exhibited current values of 12 and 0.5 µA with semiconducting behavior. f) Electronic band gaps were calculated from *R* versus *T* measurement, highest bandgap was obtained for the GBNS sample of 0.96 eV compared to 0.83 eV for GBNM and 0.69 eV for the GBNH sample. g,h) Gas sensing behavior of hybridized RGO‐RBNO sample was studied upon exposure of analyte ammonia gas at different concentrations (70 and 100 PPM). Its repeatability behavior was demonstrated for five cycles at 70 PPM. i) Straintronics behavior was recorded for hybridized RGO‐RBNO samples (10, 20, and 50) wt% for spin‐coated on PET substrate, 50% RGO in BN sample exhibited the highest current from 2 to 17 µA when strain changed from 0% to 0.16%. Optical and SEM images of the device used for electrical measurements are shown in the top panel.

Electronic behavior is expected to change upon hybridization. Bandgap shrinking of BN (6.1 eV) toward visible bandgap (i.e., semiconductors) is expected. Therefore, it is necessary to investigate their electrical properties. Hybridization induces the localized electronic state in BN; thus, its existing electronic states get modified, and hence new states will be rendered in the BN system. *I*–*V* measurement was performed by two probes by applying a voltage from −2 to 2 V on the hybridized sample (by heating method) deposited on finger‐electrode coated (top panel in **Figure** **5**) polyethylene terephthalate (PET) substrate using spin coating with different wt% of RGO (10% to 50%) (see Figure 5a). Less % of RGO in the hybridized sample exhibited insulating behavior, but the amount of RGO wt% increased from 10 to 50 in the hybridized sample; the current value significantly increased and overall behavior became like RGO. The effect of strain on electronic properties of the hybridized sample (with different wt% of RGO on RBNO) was also studied by performing (*I*–*V*) measurement under different compressive strain conditions in voltage range −2 to 2 V (see Figure 5i). For 10 wt% RGO‐RBNO hybridized system, current value changed from 0.18 to 0.35 µA when strain % increased from 0% to 0.16%. Similarly, for the 20% RGO‐RBNO system, the current value changed from 1.8 to 19 µA, and for the 50% RGO‐RBNO system, it turns out that the current value changed significantly from 17 to 25 µA when strain % changed from 0% to 0.16%. Such significant changes observed in *I*–*V* characteristics under strain conditions are caused by interlayer coupling and changes in their interlayer distance and strain‐induced electronic band gaps.

Electrical properties of hybridized (50:50) wt% of RBNO‐RGO samples by various methods (GBNH, GBNS, and GBNM samples named as 1, 2, and 3 in the measurement) were compared by measuring their in‐plane (Figure 5b,c) and out‐of‐plane (Figure 5d,e) by measuring *I*–*V* and photocurrent response of hybrid material. GBNM hybrid sample exhibited the highest in‐plane current value, 65 µA, and semiconductor behavior compared to 40 µA for GBNS and 35 µA for GBNH. When photocurrent measurement was performed in the hybridized sample, GBNS exhibited the highest photocurrent of 46 µA upon exposure to the light source compared to GBNM and GBNH samples. Such photo‐response of the solvothermal hybridized sample (GBNS) hints at the semiconductor nature highly, with substantial modification of Dirac point from sharp conic nature to parabolic nature at Γ‐point due to strong interlayer coupling and bond formation and as an effect bandgap opens. To validate the argument, we performed the *R* versus *T* measurement for hybridized samples (see Figure 5f and details in Figure S19, Supporting Information). The highest electrical bandgap 0.96 eV was obtained for a solvothermal hybridized (GBNS) sample compared to 0.69 eV for GBNH and 0.83 eV for the GBNM sample.

To figure out the modulation of charge carrier concentration along with the type of semiconductor, we performed the Hall effect measurement for (GBNH, GBNS, and GBNM named as N, H, and Mi in the measurement) samples (see details in Figure S21, Supporting Information). Carrier concentration calculated for GBNH was 1.8 × 10^16^ C m^−2^ for GBNS; it was found 3.9 × 10^14^ C m^−2^ and GBNM exhibited 1.89 × 10^15^ C m^−2^. Point to be noted is that GBNH and GBNS exhibited n‐type semiconducting behavior. In contrast, GBNM exhibited p‐type behavior. Hybridized material explored for gas sensing application (see Figure 5g) for detecting toxic NH_3_ gas due to its industrial significance. Hybridized material exhibited an excellent response to NH_3_ gaseous molecules when exposed to the testing material at room temperature due to chemisorption and the reducing nature of NH_3_ molecules in the hybrid materials. Its electrical resistance value decreased upon the exposure of analyte gas molecules and regained its original resistance value when exposure is turned OFF. We have tested different concentrations (70 and 100 ppm) of NH_3_ gaseous molecules, and response times of 45 s and recovery time of 50 s have been recorded. We also checked for repeatability of our sensor for four cycles with the same concentration (70 ppm) of analyte NH_3_ gas and found good repeatability.

Hybrid structures containing stacking of dissimilar 2D materials Gr‐BN layers are in great interest for inelastic electron tunneling devices and resonant tunnel diode applications. Stacking such materials induces band gap in graphene and provides less scattering and dangling bond‐free surface for device application in contrast to SiC and SiO_2_ surfaces (with higher flexibility). Tunneling current measurement will be interesting to investigate in the hybridized system. Therefore, we performed *I*–*V* measurements on hybridized RGO‐RBNO sample (hybridized by microwave GBNM and solvothermal GBNS method) in the voltage range −2 to 2 V in the presence of light and without light (see Figure 5d,e). In both cases, we observed nonlinearity in *I*–*V* measurements with negative breakdown voltage −1.8 V in the case of the microwave‐hybridized sample (GBNM) with a slight dip in exponential behavior in forward bias (asymmetry), due to quantum state which was not present in hydrothermal hybridized (GBNS) sample.

**Figure 6:**
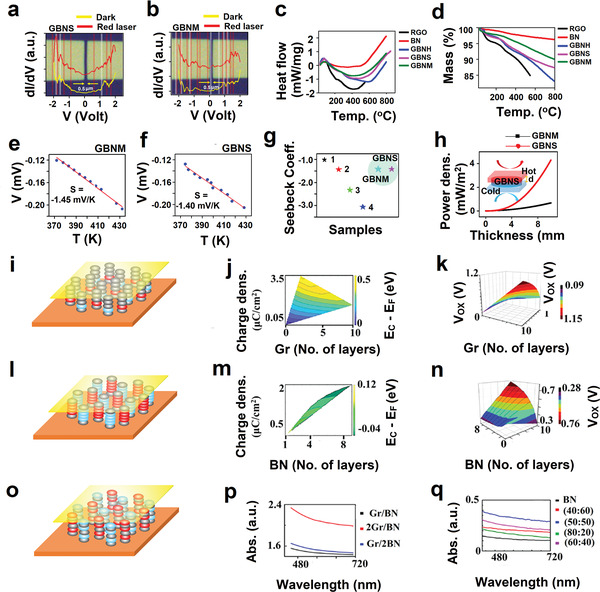
Optical image of the electrodes having separation of 0.5 µm and its quantum state measurements in the dark and with excitation of red laser light for a) GBNS and b) GBNM. c) DSC measurements for GBNS, GBNM, GBNH, RGO, and BN. d) TGA measurements for GBNS, GBNM, GBNH, RGO, and BN. e,f) Thermal conductivity measurements for GBNM and GBNS. g) Seebeck coefficient comparison of GBNS and GBNM with various samples 1‐pristine ϒ‐graphyne, 2‐ϒ graphyne with BN at the chain, 3‐ϒ graphyne with BN at the ring, and 4‐ϒ graphyne like BN sheets.^[^ ^59^ ^]^ h) Simulation result of thermoelectric output power density with the variation of thickness of coating materials for GBNS and GBNM hybrid materials. i) Schematic diagram of Au/Gr/BN/Gr/Au device structure for tunneling current measurement, where Gr layer varies from 1 to 10 layer. j) Simulated charge density and energy difference between *E*_c_ and *E*_F_ (*E*_C_−*E*_F_ (eV)) in hybrid Au/Gr/BN/Gr/Au system by changing the number of Gr layers from monolayer to 10 layers, carrier density decreased from 3.82 to 1.56 µC cm^−^2 and the energy difference between *E*_C_ and *E*_F_ decreased from 0.56 to 0.36 eV. k) The potential drop across dielectric (*V*_ox_) for interface fell from 0.42 to 0.1 V, with increasing Gr layer from monolayer to 10 layers. l) Schematic diagram of tunneling device Au/Gr/BN/Gr/Au structure for tunneling current measurement, where BN layer varies from 1 to 10 layers. m) Varying layer numbers, monolayer to 10 layers of BN charge carrier density increased from 0.008 to 2.2 µC cm^−^2 and the energy difference between *E*_C_ and *E*_F_ (*E*_C_−*E*_F_ (eV)) decreased from 0.12 to −0.06 eV. n) The potential drop across the dielectric layer increased from 0.2 to 0.76 V by changing the BN layer number (1–10). o) Schematic diagram of Gr/BN/Gr, BN/Gr/BN used for UV‐vis spectra simulation p) simulated UV‐Visible spectra for Gr/BN with varying numbers of the layer. q) UV‐vis spectra for microwave‐hybridized RGO‐RBNO samples with different wt% of RGO on RBNO and compared with simulation results.

We then fabricated two‐terminal device with electrode separation of ≈500 nm, using extreme ultraviolet lithography. Such electrode separation is supposed to minimize grain boundary contribution in the observed data and hence minimize noises. In conductance measurement at room temperature, with and without red laser light, in fact we observed quantized conductance peaks (coupled quantum states), i.e., signature of interlayer coupling in G‐BN systems, i.e., in GBNH and GBNM. In both the samples, shifting of the quantum states was observed under the exposure of red laser light as compared to those without light. This is due to the modulation of interlayer coupling between graphene and BN mediated by red laser exposure. Also, new quantum states arise due to excited states being accessed (see **Figure** **6**a,b). The differential scanning calorimetry (DSC) and thermogravimetric analysis (TGA) measurements were performed for specific heat calculation and thermal stability of the materials (see Figure 6c,d). We found the *C*_p_ values for GBNH 0.15 J g^−1^ °C^−1^, GBNS 0.10 J g^−1^ °C^−1^, GBNM 0.06 J g^−1^ °C^−1^, RGO 0.12 J g^−1^ °C^−1^, and BN 0.04 J g^−1^ °C^−1^ (see Figure S27, Supporting Information).

Graphene is gapless semimetal, with high mobility of ≈10^6^ cm^2^ V^−1^ s^−1^ and high thermal conductivity of ≈1324 W m^–1^ K^–1^ but has minimal intrinsic charge carrier concentration of ≈8.75 × 10^12^ cm^–2^. In contrast, BN is an insulator with a wide band gap of ≈6.1 eV, and charge carrier concentration of ≈3.53 × 10^14^ cm^–2^, and thermal conductivity of 751 W m^–1^ K^–1^. The hybrid structure of graphene‐BN will be very interesting for thermoelectric application due to the possibility of modulation of charge carrier concentration, band gap, and thermal conductivity. Therefore, we measured the thermoelectric measurements for GBNS and GBNM samples and found that Seebeck coefficients were −1.40 and −1.45 mV K^−1^ (see Figure 6e,f). The negative sign in the Seebeck coefficient signifies the n‐type of the materials. We also compared the Seebeck coefficient of different graphene‐BN hybrid systems with experimental results obtained for GBNS and GBNM. We found that our GBNS and GBNM exhibit similar behavior (see Figure 6g). Also, we compared the simulated thermoelectric output power density with variation in thickness for GBNS and GBNM and found high output power density for the GBNS system (see Figure 6h) due to higher mobility of charge carrier compared to GBNM.

For further insight into the charge transport, charge density and interfacepotential, and alignment of bands, we performed a simulation of *I*–*V* measurement for Au/Gr/BN/Gr/Au system for 2 V in two cases: 1) BN layer number starting from monolayer to eight layers and 2) Gr layer from monolayer to nine layers. When graphene layer number increased from monolayer to nine layers in Au/Gr/BN/Gr/Au sequence, as shown in schematics of Figure 6i, the charge density decreased from 3.82 to 1.56 µC cm^−2^ and the difference between (*E*_C_−*E*_F_) eV decreased from 0.56 to 0.36 eV due to the cumulative effect of the number of layers and applied voltage (see Figure 6j). Monolayer BN in (Gr/BN/Gr) structure acts as the best tunneling barrier for charge carriers. When voltage was applied across the device, induced charge (mostly) gets drained out in another end because of the lower (*E*_C_−*E*_F_) energy barrier.

Similarly, the potential drop across dielectric (*V*_ox_) for interface decreases from 0.42 to 0.1 V with increasing Gr layer from monolayer to eight layers (Figure 6k). When the BN layer increased from monolayer to eight layers in device structure Au/Gr/BN/Gr/Au, as shown in schematics in Figure 6l, we observed a contrasting behavior; charge density increased from 0.008 to 2.20 µC cm^−^2, and the energy difference between *E*_C_ and *E*_F_ (*E*_C_−*E*_F_ (eV)) decreased from 0.12 eV to minimal that is because of BN layer which acts as active barrier layer responsible for charge transport with an increasing number of layer, the less number of charge carrier can sieve through the BN as an effect; the interface charge carrier density gets enhanced (see Figure 6m). Also shift in the potential barrier is much higher than the conduction band energy for the second interface. The potential drop across dielectric (*V*_ox_) for interface increased for increasing number of BN layer (from monolayer to nine layers) from 0.2 to 0.76 V which signified that mostly blocking of the charge carrier on the second interface, as an effect the Columbic potential increased (see Figure 6n). The optical properties of such hybridized system Gr‐BN will be interesting to measure because of the possibility of opening bandgap in the visible range. Therefore, we performed UV‐vis absorption spectra for microwave‐hybridized RGO‐RBNO samples with different wt% of RGO on RBNO (see Figure 6o) and simulated it for Gr/BN system with the varying number of layers (see Figure 6p) as shown in schematic in Figure 6q, and found a small hump in ≈620 nm range for RGO‐RBNO (50:50) wt%, almost matching with simulated result (two layers of Gr‐one layer of BN) system. There might be a change of stacking two layers RGO‐one layer RBNO in the dynamic condition of field and pressure.

Apart from the sequence design using dilute supernatants of individual sheets, which ultimately leads to bandgap engineering as reported earlier,^[^ ^45^ ^]^ the extreme electric field ≈10^6^ V cm^−1^ in microwave treatment, and extreme pressure ≈1 MPa and thermal conditions ≈200 °C in solvothermal treatment are responsible for effectively bringing layers close to each other and help for the formation of chemical bonding in constitute layers (Sp^2^+Sp^3^), much like conversion of graphite to graphite‐like diamond's structure at elevated pressure and temperature, also modulating twist angles as well as giving rise to shear among the planes. In fact, the microwave has been employed for the synthesis of 2D metal oxides and 2D ferrites, where the electric field in microwave breaks the bonds in precursors and makes new ones which eventually helps in attaining direct crystallization of new materials systems.^[^ ^46^, ^47^, ^48^ ^]^ The microwave irradiation of highly absorbing materials such as graphene results in extreme energy density under an ambient environment. It generates very intense visible (reddish yellow) plasma (Figure 3a). In general, focused intense laser or extreme electric field also creates high energy density conditions where the visible spark can be observed which results in instant bond dissociation or bond formation.^[^ ^49^, ^50^, ^51^ ^]^ Under intense plasma, defect formation, defect cluster formation, defect movement, and physical migration of atoms via electromigration or thermomigration take place. The use of such intense electric/electromagnetic fields therefore still remains very exciting for the synthesis of materials via nonequilibrium pathways. The whole process occurring in a few seconds under plasma processing does not provide the system sufficient time to respond to the external stimuli (energy source) and the effect so brought is irreversible. All these effects brought by applied external fields will determine coupling strength between the layers. When coupling strength exceeds a threshold, chemical bonds will form, and exactly the same has been observed in the present research. Interlayer distance, twisting angle, strain, individual materials, and remnant functional groups are among several parameters which would determine coupling strength. 2D materials are atomically thin and their behavior is quantum in nature, interlayer‐coupled 2D materials exhibit exotic quantum states, called pairing states.

Pairing term in Hamiltonian can be given by^[^ ^52^ ^]^

(1) $$H=\left[\begin{array}{cc} H^{1}\left(r,p\right) & S\left(r,p\right)\\ S{\left(r,p\right)}^{†} & H^{2}\left(r,p\right) \end{array}\right]$$

where 𝐻**^𝑖^** (𝑟) is generalized Hamiltonian, and 𝑆 (𝑟, 𝑝) is interacting (interlayer coupling term)

(2) $${\left\{S\left(r,p\right)\right\}}_{\alpha ,\beta }=\frac{1}{V_{\mathrm{UC}}}\;M_{i\alpha \beta }e^{-iG_{i}.\Delta U\left(r\right)}t_{\alpha \beta }\left(K_{i}+p\right)$$

where 𝑉*_𝑈𝐶_* represents the volume of the unit cell and ∆(𝑟) represents the deformation field due to a change in the local stacking order, 𝐾_𝑖_ represents the translation vector in momenta space and 𝛼 𝑎𝑛𝑑 𝛽 represents the basis atom in layers. The ∆(𝑟) plays a major role to decide the interlayer coupling energy contribution to the Hamiltonian and depends upon the external parameters (pressure, electric field, temperature, and orientation). It is these quantum states in bilayer graphene, or twisted Gr‐BN bilayer, and even in several heterolayered systems, which enable these advanced materials very special character such as superconductivity.^[^ ^53^, ^54^ ^]^ In the new era of emerging technologies, quantum information processing and storage are going to have a crucial role. External parameter tunable quantum states in coupled heterolayered 2D materials (i.e., 2+*δ*‐dimensional materials) systems can do wonders in this un‐walked territory, it is proposed.

In conclusion, the discovery of a new class of advanced materials systems termed 2+*δ*‐dimensional materials is reported. These novel materials were synthesized employing extreme electric field and thermal conditions in the microwave or extreme pressure and thermal condition in solvothermal reactions. Out‐of‐plane chemical bond formation between component layers has been witnessed through XPS signals corresponding to C—B and C—N bonds in G‐BN systems, Mo—N and Mo—B bonds in MoS_2_‐BN systems, etc. Distinct vibrational Raman spectroscopic fingerprints, completely different from individual component layers (2D materials), e.g., 1320 cm^−1^ for the G‐BN system and 345 cm^−1^ for the MoS_2_‐BN system have been observed. The interlayer distance reduces with the increase in electric field in microwave treatment and effective pressure in solvothermal treatment. When interlayer distance matches the theoretical equilibrium interlayer distance, chemical bond forms. The charge density difference profile for the G‐BN stacking system at an interlayer distance of 2.7 Å reveals apparent charge transfer as well as P_z_—P_z_ chemical bond formation between two constituent layers arising from the strong interlayer coupling. While bandgap tunability has widely been attained across hybrid combinations, this work discovered the presence of mid‐gaps and diode behavior exhibited by the presented G‐BN hybrid systems for the first time. MoS_2_‐BN hybrid systems at equilibrium distance, considering MoS_2_ to be semiconducting with the direct bandgap of 1.98 eV, after the processing, the effective bandgap was turned to be 2.7 eV at equilibrium distance of 3.3 Å and 2.9 eV at 2.7 Å, indicating the formation of 1T metallic phase due to exotic conditions of processing. Molecular dynamics simulation of the thermo‐pressure or thermo‐field exotic processes occurring inside the reaction vessel hints at local contacts (or, z‐pinches) between the component layers. The presented hybrid systems were explored for their potential applications to detect ammonia gas, strain sensing, and photodetectors. The present discovery will have far‐reaching consequences in the development of such novel systems and emerging generations of devices and sensors.

## 3 Experimental Section

### Synthesis of 2D Materials and Their Hybrids by Sonochemical Method

Graphene and BN sheets were exfoliated from their bulk precursors (graphite and HBN) employing ultrasonication (20 kHz) with the addition of adequate solvent (isopropyl alcohol, IPA) in a 250 mL beaker along with 1 g of each material (graphite and BN) and sonicated for 48 h. After sonication, the supernatant of graphene and BN were then mixed by taking wt% of graphene (0.5 to 5) in BN and further sonicated for 4 h. Resultant hybrid materials were used for various characterizations.

### Synthesis of 2D Materials by Modified Hummer's Method

Modified Hummer's method was employed for synthesizing 2D sheets of (Gr, BN), but for synthesizing 2D sheets of MoS_2_ and WS_2_, the protocol was modified by tuning the wt% of initial precursors (1:4) wt% of MoS_2_ and KMnO_4_ as well as for WS_2_ with KMnO_4_. Initially, MoS_2_ and KMnO_4_ mixture was ground in a mortar‐pestle for homogenous mixture; the powdered mixture was kept inside the air‐tight container. The beaker containing an acid solution having (1:9 vol%) of (H_3_PO_4_:H_2_SO_4_) was kept inside the freezer for 4 h to sufficiently cool down. Then the powdered precursor mixture was slowly poured down into the acidic solution. After that, the mixed solution was placed over a magnetic stirrer cum hot plate at 65 °C for 3 h under 300 rpm. When the process was completed, it was allowed to cool down at room temperature. After that, H_2_O_2_ solution ≈3 mL was added to the solution to terminate the reaction. Extra ice cubes were added to the terminated reaction to make an even lower temperature. Finally, purification was realized by adequate washing and cleaning of reaction products formed inside the vessel. Cleaning processes involved centrifugation using various solvents in the sequence deionized (DI) water, HCl, IPA, HCl, IPA, and DI water at 10 KRPM for 5 min. A similar protocol was employed for exfoliating WS_2_ sheets.^[^ ^55^ ^]^

### Synthesis of Hybrid Material of (RBN‐RGO) and RBNO‐MOS_2_ and MoS_2_ with WS_2_

Synthesis of hybrid materials required pre‐reduction of chemically exfoliated 2D sheets (GO, BNO, MoS_2_, and WS_2_) using solvothermal processes individually. 1 g of GO, BNO, MoS_2_, and WS_2_ was separately placed in a Teflon lined cup (100 mL) containing reducing solvent DMF (50 mL). The reduction process was done for 4 h and then allowed to cool down at room temperature. The obtained product was dried at 300 °C to evaporate the solvent for 3 h. Obtained powders were RBNO, RGO, MoS_2_, and WS_2_. After reduction, obtained powder samples were hybridized by three processes: heating, solvothermal, and microwave. For the RBNO‐RGO hybrid initially, RBNO and RGO were mixed in (1:1) wt% in 100 mL DMF solvent and was sonicated for 3 h. The obtained solution was divided into two parts; one portion (50 mL) was treated by the solvothermal process in a Teflon‐lined autoclave at 200 °C for 4 h. After completing the process, it was allowed to cool down at ambient temperature, and the remnant solvent was evaporated by heating at 300 °C for 2 h. After drying, the obtained product was RGO‐RBNO (called GBNS henceforth) hybridized. The second half of the 50 mL solution was transferred in a sealed tube (100 mL) and placed inside the household microwave oven (2.45 GHz) by setting microwave power at 720 W and exposed for 5 min in a pulsed manner. After each pulse, the sealed tube was opened to release pressure and cool down at room temperature. These steps were repeated until 5 min. After the remaining solvent was evaporated by heating at 200 °C for 3 h, the obtained solid product was a microwave‐hybridized RGO‐RBNO (called GBNM henceforth) sample. Similarly, the heating method was employed for attaining RBNO‐RGO hybrid, taking wt% (1:1) in DMF solvent and sonicated it for 4 h. Homogenized mixed product was heated for 3 h in the hot plate for evaporating the solvent; the resultant powered mixture was hybridized RBNO‐RGO (called GBNH henceforth). Likewise, the same procedures (heating, solvothermal, and microwave) were followed for hybridizing RBNO‐MoS_2_ and MOS_2_‐WS_2_ samples.

### Microscopy and Spectroscopy

TEM from JEOL (JEM‐2100) was used to study the morphology of sheets in hybrid systems (RGO‐ RBNO, RBNO‐MOS_2_, MoS_2_‐WS_2,_ and graphene‐BN). HRTEM images were acquired by using Titan Themis 300 kV from FEI for graphene‐BN, RGO‐RBNO, RBNO‐MoS_2_, and MoS_2_‐WS_2_ systems. Raman spectra were obtained for hybrids (RGO‐RBNO, RBNO‐MoS_2_, MoS_2_‐WS_2_, and graphene‐BN) utilizing a Micro‐Raman spectrometer from (STC Japan) with He‐Ne (633 nm) laser as an exciting source by STR Raman spectrograph. An optical microscope (Olympus) was used for optical image and zooming purposes, having magnification up to 100X for focusing laser spot. Electric field‐dependent Raman spectra were attained on the hybrid sample RGO‐RBNO by applying DC voltage. Chemical purity and bond formation of synthesized hybrid samples (RGO‐RBNO, RBNO‐MOS_2_, and MoS_2_‐WS_2_) were analyzed with ESCA+ Omicron nanotechnology under extremely high vacuum conditions (10^–12^ mbar). FTIR was also used as a supporting diagnosis tool for chemical purity and physical bond formation for hybrids employing (spectrum one) Perkin Elmer system having rage (400–4000 cm^−1^) in solid form.

### Electrical and Optoelectronic Measurements

*I*–*V* with photocurrent measurements was performed using a two‐probe method using Keithley (2400) as a source meter interface with a computer using a lab view program.

### Hall Effect Measurement

Hall effect measurements were performed for hybrid systems RGO‐RBNO achieved by three different methods. Initially, hybrids powder samples were taken and palletized (10 mm) using KBr palletizer (hydraulic) by applying 1.5 ton pressure. The obtained pallet was used for Hall effect measurement. Keithley's (2400) source meter was used to measure voltage and current. A digital Gauss meter was used for measuring the magnetic field and a DC power supply source for supplying fixed current to the electromagnets. Four probes were used for contact with the sample. Prior to placing the sample, magnetic field offset was performed to ensure no error in measurement. Fixed magnetic field 2500 Gauss was applied by electromagnets connected to the AC power supply source. The sample was kept between two electromagnets, and the corresponding voltage was measured by Keithley 2400. The same measurement was repeated by reversing the polarity of the probe, and measurement was performed with the same current across the sample. The average value of *V*_H_ was calculated by using formulas

(3) $$V_{H}=\frac{|V_{1}+V_{2}+V_{3}+V_{4}|}{4}$$

Resistance was measured by the van der‐Pauw method. Likewise, resistivity was calculated by the below‐mentioned formula

(4) $$\rho =\frac{\pi }{\ln2}\left(f_{a}*t\right)\frac{\left(R_{1}+R_{2}\right)}{2}$$

where *ρ* is the resistivity of the sample, *f*_a_ is a geometrical factor (for the spherical case, it is 1), and *t* is the thickness of the sample. The thickness of the sample was measured using the Screw gauge.

### Gas Sensing Measurement

RGO‐RBNO sample (50:50) wt% was spin‐coated at 3000 rpm on PET substrate and was dried at 100 °C on a hot plate. After drying, electrical contacts were made with silver paste and copper wire and then were placed over a hot plate to evaporate epoxy for minimal contact resistance. The test sample was placed inside a desiccator to avoid any effect of moisture and unwanted gas. The testing chamber was degassed at 10^–3^ tors with the help of a rotary pump. Gas sensing measurements were performed on an indigenously designed chamber. All electrical contacts were connected with a computer interface via Keithley 2420 source meter in the solar cell tester (Model CT50AAA) under ambient conditions at 30 °C. Liquid ammonia (conc. 30%) analyte molecules were injected in the testing chamber using a precalibrated syringe via rubber cork. The following formula was used for converting mL into ppm

(5) $$C=\frac{22.4\rho TV_{s}}{273MV}$$

where *C* is defined as the concentration of gaseous ammonia (ppm), *T* (K) represents the temperature of the testing sample, *ρ* represents the density of liquid ammonia (g mL−1), *V*_s_ represent the volume of ammonia in liquid form (µL), *M* is the molecular weight of ammonia (g mol−1), and *V* represents the chamber volume (L). When analyte molecules of aqueous ammonia were injected in the chamber, a change was observed in the current for the RGO‐RBNO sample due to chemi‐resistive behavior where ammonia gas molecules would give an electron to the system, resulting in effective resistance would decrease.

The following formula could calculate the response of the sensor

(6) $$\frac{\Delta I}{I}=\frac{I_{{\mathrm{NH}}_{3}}-I_{o}}{I_{o}}$$

### Density Functional Theory

First principle DFT calculations were performed using local density approximation (LDA). Quantum Espresso^[^ ^56^ ^]^ was used to investigate the effect of external electric field on deformations of 50:50 composition of graphene/BN system; norm‐conserving pseudopotentials^[^ ^57^ ^]^ were defined for carbon, boron, and nitrogen to define the core–valence interactions. A 36 × 36 × 1 Monk horst‐ Pack k point mesh was used to perform the self‐consistent calculations. The vacuum region was set to be ≈20 Å in the *Z*‐direction to avoid interaction during periodicity. Geometric optimization was performed until the residual forces on atoms were less than 0.01 eV A^−1^. The electric field of 9 × 10 6 V m^−1^ was applied in the perpendicular direction to the 2D slab of graphene‐BN lattice along with dipole correction.^[^ ^58^ ^]^ According to these calculations, the distance between the optimized layers after the application of the electric field was found to be 3.501 Å.

### Thermoelectric Measurements and Their Simulation

Thermoelectric measurements were performed by the differential method where one end of the sample was heated, and the other was kept at room temperature to have a temperature gradient Δ*T*. The thermoelectromotive force Δ*V* was measured with respect to the temperature difference between the hot and cold end. The Seebeck coefficients were obtained for GBNM and GBNS samples by plotting graphs between the attained voltage *V* (Thermo emf) and the temperature of the samples. The thermoelectric output power density simulation was done in open source nanoHub by taking a thermoelectric generator module with convective heat transfer where contact resistance of the electrodes, thermal conductivity, Seebeck coefficient, electrical conductivity, thickness of the material, and hot end and cold end temperature parameters were supplied.

## Conflict of Interest

The authors declare no conflict of interest.

## Author Contributions

P.K. conceived the idea. T.K.S. carried out the synthesis of 2D materials hybrids and explored its applications. M.M. carried out theoretical modeling works for the hybrid system under the supervision of G.J.C. A.B. performed DFT calculation and band structures of 2D hybrids. N.K. helped with HRTEM microscopy. P.K. discussed results with G.J.C. T.K.S., P.K., M.M., and G.J.C. wrote the manuscript together. The project was overseen by P.K. and G.J.C.

## Supporting information

Supporting Information

## Data Availability

The data that support the findings of this study are available from the corresponding author upon reasonable request.
