# Supplementary material for: 2+δ‐Dimensional Materials via Atomistic Z‐Welding
Source: Adv Sci (Weinh). 2022 Sep 11;9(32):2202695. doi: 10.1002/advs.202202695 (PMC9661819; doi:10.1002/advs.202202695)
Supplement: Supplementary file 1 — Supporting Information [file ADVS-9-2202695-s001.pdf]

ADVANCED SCIENCE
Open Access

Supporting Information

for *Adv. Sci.*, DOI 10.1002/advs.202202695

2+δ-Dimensional Materials via Atomistic Z-Welding

Tumesh Kumar Sahu, Maithilee Motlag, Arkamita Bandyopadhyay, Nishant Kumar, Gary J. Cheng and Prashant Kumar**

2+σ dimensional materials via atomistic Z-welding

Tumesh Kumar Sahu[1,2] , *Maithilee Motlag*[3], *Arkamita Bandyopadhyay*[4], *Nishant Kumar*[1], *Gary J. Cheng*[3,5,6,*], *Prashant Kumar*[1,6,7,]*

[1]Department of Physics, Indian Institute of Technology Patna, Bihta Campus, India-801106.
[2]Department of Physics, Shri Ramdeo Baba collage of engineering and Management, Nagpur Maharashtra, India-440013
[3]School of Industrial Engineering, Purdue University, West Lafayette, Indiana, USA- 47907.
[4]Bremen Center for Computational Materials Science, Eingang A, 28359 Bremen, Germany.
[5]School of Materials Engineering, Purdue University, West Lafayette, Indiana, USA- 47907.
[6]Birck Nanotechnology Centre, Purdue University, West Lafayette, Indiana, USA- 47907.
7Global Innovation Centre for Advanced Nanomaterials, The University of Newcastle, Australia-2308

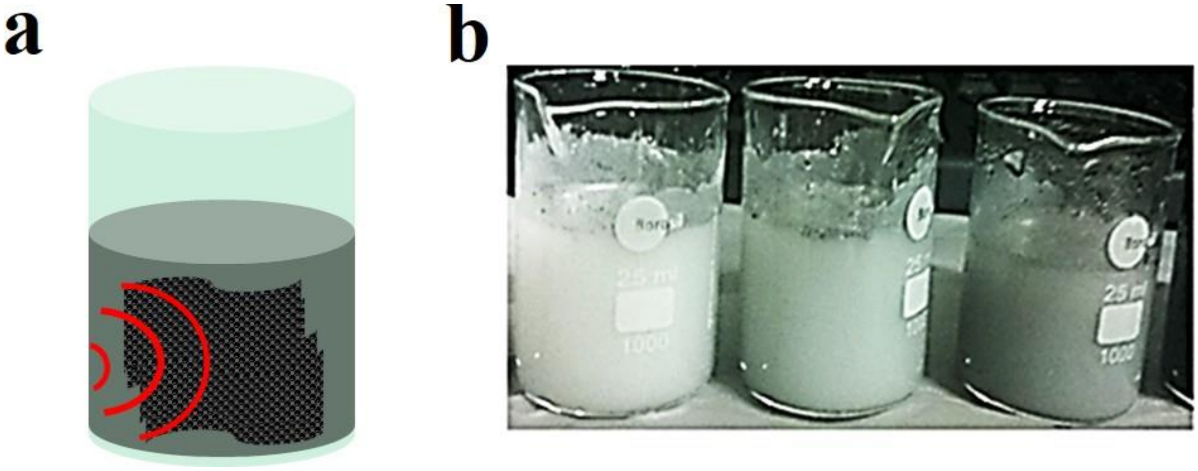

Fig. S1 (a) Schematic diagram of sonochemical exfoliation processes for graphene sheets in IPA solution. (b) Camera image of a sonochemical hybridized sample of Gr-BN with different wt. % (0.5 to 1) from left to right.

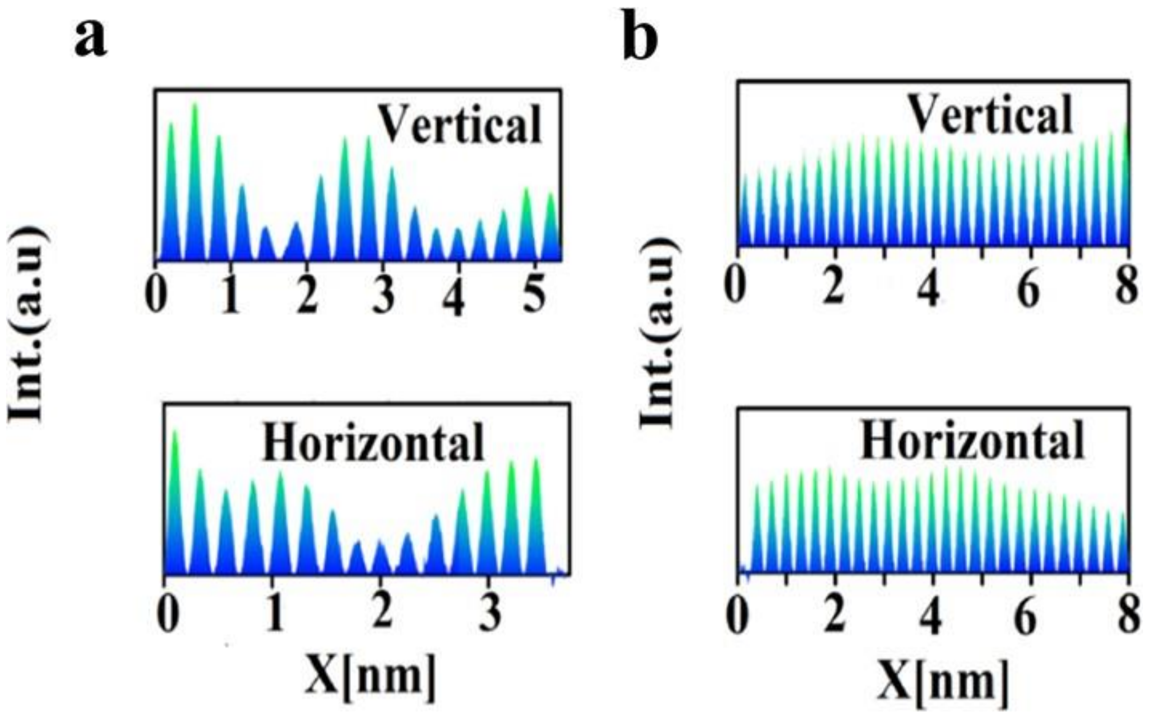

Fig. S2 Atomic profile for hybridized Gr-BN samples obtained from HRTEM, measured along with atomic arrays of atoms (horizontal) and across the atomic arrays of atoms (vertical) for (a) 1 wt. % and (b) 0.5 wt. %.

Section1: XRD details of RGO-RBNO, MoS_2-WS_2, and MoS_2-RBNO system

A hybridized sample of RGO-RBNO was studied using the X-ray diffraction technique (XRD) to have a glimpse of inter-layer coupling and find out new hybridized peaks due to C-B and C-N bond formation. When XRD measurement was performed on the GBNH sample, we observed 2θº peaks at 26.5º, 41.4º, 43.6º, 54.9º, 59.4º, 71.2º, and 75.8º. Similarly, GBNS exhibited XRD peaks at 26.7º, 41.4º, 43.9º, 56.1º, 59.5º, 71.3º, and 75.9º. When microwave power was used as an exotic condition (temperature and electric field) for hybridizing and obtained product was diagnosed with XRD measurement, it exhibited peaks at 26.60º, 41.4º, 43.7º, and 55.0º, respectively 59.7º, 71.3º, and 75.7º. XRD peak at~ 26.0º of B.N. corresponds to {002} facet of hexagonal closed pack structure shifted from their original position 0.5º for GBNH, 0.7º for GBNS, and 0.6º for GBNM sample. We observed a change in inter-layer d-spacing from ~3.42 Å to 3.3 Å for the GBNM sample, which was ~2.1 % less than their original d-spacing. XRD is a bulk characterization technique that gives information at a macro level; any minute changes in the micro-level will not be detected by XRD. However, the Shift in micron level is moderately significant; we expect more changes would have occurred at the local atomic level, and M.D. simulation results support the same; the local distance waschangedfrom3.3Åto2.7Åatthe

Hybridized location. We also have found a distinct XRD peak at 54.9º due to diffraction from localized B_4C Like- crystal structure formation upon hybridization between constitute layers[3, 23-24].

A similar hybridization technique was employed for semiconductor MoS_2 and insulator RBNO sheets, which have contrasts in their electronic and optical behaviors. The hybridized sample is expected to exhibit bandgap tuning, charge carrier density enhancement, and the emergence of new bond formation (Mo-N and Mo-B) upon hybridization. When XRD was used to examine the heating method's hybridized sample, various peaks were observed at 14.2º, 28.7º, 32.5º, 33.4º, 35.8º, 39.4º, and 43.8º, respectively 49.6º, 55.0º, and 59.7º. A similar measurement was performed for the solvothermally hybridized sample; it exhibited peaks at 14.3º, 26.6º, 28.9º, 32.6º, 33.4º,35.8º, and 39.4º, respectively 44.0º, 49.7º, 55.0º, and 56.0º. whereas, microwave treated hybrid sample demonstrated peaks at 14.4º, 26.8º, 29.0º, 32.7º, 33.5º, 35.9º, 39.5º, 44.1º, 49.8º, 55.1º, 56.1º, and60.1º.Interestingly peak at 13.9º of {002} facet of the hexagonal MoS_2 phase shifted upon hybridization. The observed shift in the peak was 2.1 % for heating and 2.8 % for solvothermal, and 3.5 % for microwave-hybridized samples. The maximum change observed in inter-layer d-spacing was 6.14Å for the microwave-hybridized sample compared to 6.3 Å for pure 2H MoS_2. We also have witnessed distinct diffraction peaks at~34.5º and 60.1º due to the Mo_2B like-structure. Similarly, peaks at ~39.4º and 44.0º signified the diffraction peaks due to MoN like-structure. The presence of various bond (Mo-N and Mo-B) in XRD suggest strong hybridization between MoS_2 and B.N. layers under microwave and solvothermal processing condition[2,5-7].

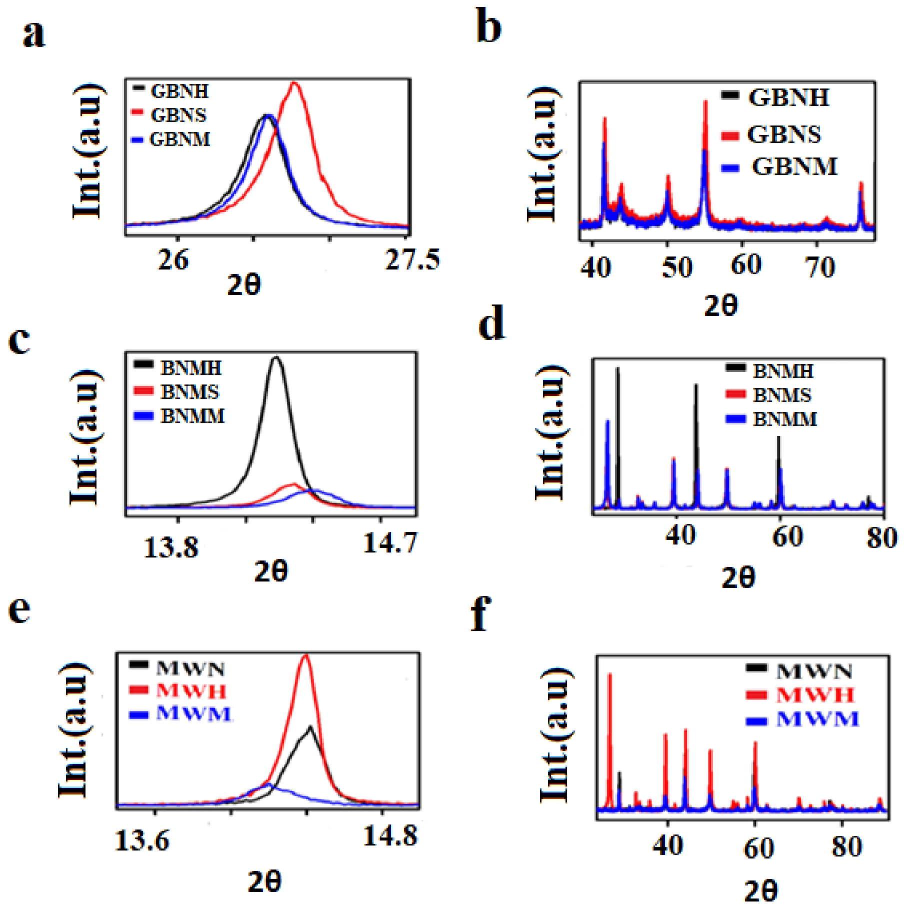

Fig. S3 XRD pattern (a) small scale scan for GBNH, GBNS, and GBNM samples and (b) large range scan for GBNH, GBNS, and GBNM samples. (c-d) Small scale and extensive scan range for MBNH, MBNS, and MBNM samples. (e-f) small scale and extensive scan range for MWH, MWS, and MWM samples.

MoS_2 and WS_2 being semiconductors, with bandgap (1.4 to 1.9 eV), both exhibit direct band gaps only when synthesized in monolayer. Synthesizing the monolayer of MoS_2 and WS_2 using scotch tape and sonication is a cumbersome process, whereas CVD is expensive and non-scalable. Overcoming such hurdles, we combine these two-dimensional materials to tune their excitonic behaviors by hybridizations (heating, solvothermal, and microwave). XRD measurement was performed for hybridized (MoS_2-WS_2) sample by MWH exhibited peaks at 14.4°, 28.9°, 31.2°, 32.7°, 33.5°, 35.8°, 39.5°, 43.9°, 49.8°, 56.0°, 58.4°, 59.8°, 62.7°, 70.2°, 73.0°, and 80.1°. Likewise, for the MWS sample, it exhibited peaks at 14.3°, 26.6°,29.0°, 31.4°, 32.6°, 33.5°, 35.8°, 39.5°, 41.6°, 44.1°, 49.8°, 50.07°, 56.0°, 58.3°, 60.1°, 62.7°, 70.1°, 72.7° and 80.2°.To distinguish microwave processing, which involves an electric field, from solvothermal hybridization (pressure and temperature), we carried out XRD measurement in the microwave process MWM sample. We observed peaks at 14.1°, 28.8°, 31.4°, 32.6°, 33.5°, 35.7°, 39.4°, 43.9°, 49.7°, 56.0°, 58.2°, 59.8°, 62.7°, 70.0°, 72.8°, and80.2°. Characteristic peak

at 14.32 º of {002} facet of WS_2(2H phase) hexagonal phase-shifted to 14.1º in the microwave-hybridized sample is also distinct peaks were observed in X-ray diffraction at 29.0º, 44.0º, and 60.1º due to hybridization between MoS_2 and WS_2. Inter-layer d- spacing was reduced to 6.2 Å in contrast to 6.3 Å for pure WS_2 slight reduction in inter-layer d- spacing was observed because of the global stacking effect. Still, at the local level, it is expected to reduce even higher.

Section 2 Raman and FTIR measurement

The 2D atomic layer constitutes atoms bound with strong covalent bonds (in-plane), whereas the out-of-plane direction exhibits van der Waals's nature. When hybridized between RBNO, RGO, MoS_2, and WS_2 is exhibited various bond formations. These bonds can be catalyzed by external perturbation by optical and electrical energy and will suffice for atomic rotational and vibrational transitions. Thus, various characteristics of vibration bond energy will be observed when diagnosed with Raman and Fourier transfer infrared (FTIR) spectroscopies. When FTIR measurement was done for GBNM sample, it exhibited vibration mode at 790, 926.9, 1088.7, 1180.9, 1238.8, 1320.1, 1483.5, 1727.6, 2313.5, 2828.0 and 2973.2 cm^{-1}. Similarly, the GBNS sample exhibited vibration modes at 752, 875, 927.7, 1096.2, 1320.9, 1494.5, 1720.0, 2539 and 2985.3 cm^{-1}. Likewise, for GBNH sample, varied rotational peaks were observed at 725.5, 903.7, 1041.2, 1269.2, 1330.4, 1447.08, 1542.88, 1725.19 and 2328.7 cm^{-1}. Typically, ~750 to the 760cm^{-1}representsanout-of-plane bending mode of B-N-B, and 1320 cm^{-1} is due to the B-N stretching mode. After hybridization, a new vibration mode at 1727.6 cm^{-1} (C-N) was observed. Similarly, a bond at (~ 1212-1270) cm^{-1} was found due to C=N vibration[13-14, 16-17]. A bond at 2928 cm^{-1} was due to symmetric stretching mode, and 2973 to 2985 cm^{-1} was due to asymmetric stretching mode of CH_3 after reducing functional group COOH to CH_3[15].

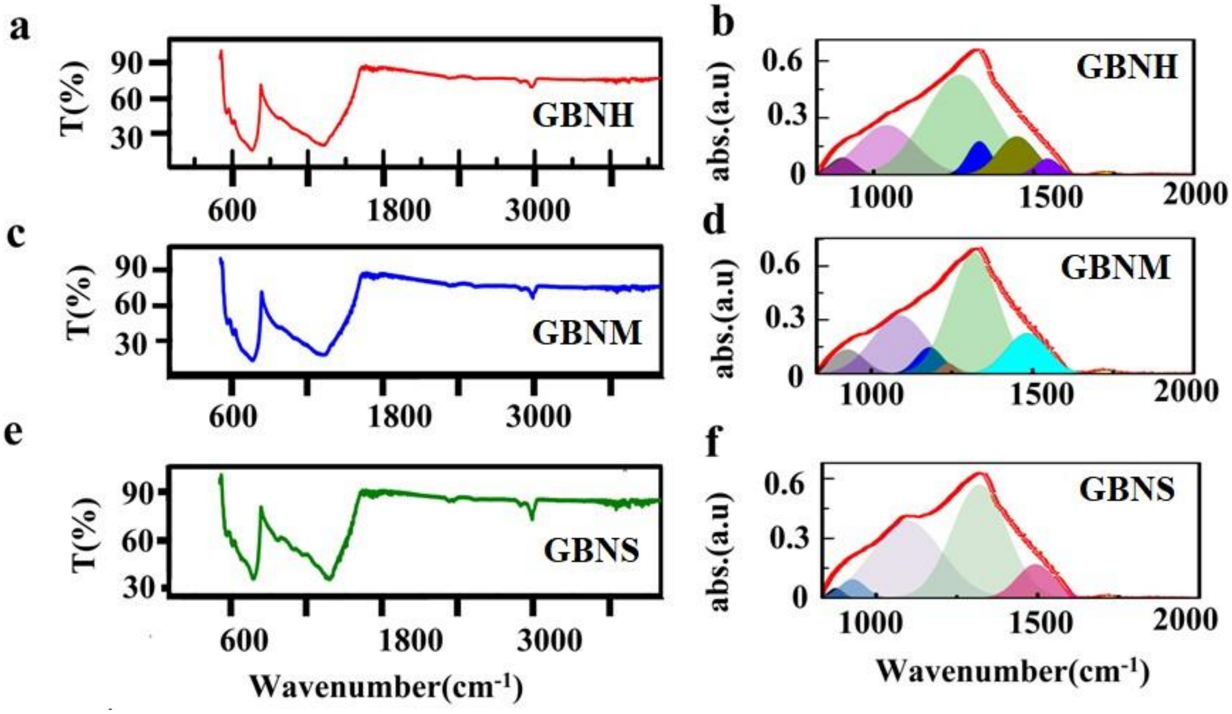

Fig. S4 (a, c, e) FTIR full range Spectra of Gr-BN hybridized (GBNH, GBNM, GBNS) samples (b, d, f) short-range spectra for (GBNH, GBNM, GBNS) samples.

Raman spectroscopic measurement was performed in the GBNH sample. After deconvolution, it exhibited Raman peaks at 1235 cm^{-1} due to hybridized (C-N) bond formation, 1322 cm^{-1} (D band), 1365 cm^{-1} due to B-N vibration mode, 1574 cm^{-1} (G band) 1599 cm^{-1} (D′ band), 2552 cm^{-1} (D+D′), 2642 cm^{-1} (2D band) and 2669 cm^{-1}. Likewise, GBNS sample exhibited Raman vibration modes at 1266 cm^{-1}, 1328 cm^{-1} (D band) and possibly due to B-C vibration mode[8-10], 1363 cm^{-1} (B-N vibration mode), 1547 cm^{-1} (G band), 1607 cm^{-1} (D′ band), 2519 cm^{-1} (D+D′), 2586 cm^{-1} (2D band) and 2702 cm^{-1}. Similarly, for the GBNM sample, in addition to pressure and temperature, a high electric field (~ 10^6 V/cm) led to the strong inter-layer coupling between (RGO-RBNO) system when it was diagnosed with Raman measurement. We observed different peaks at 1319 cm^{-1} due to (C-N)[11] bond formation 1350 cm^{-1} (B-N vibration), 1579 cm^{-1} (G band), 1603 cm^{-1} (D′), 2994 cm^{-1} (D+D′), and 2547 cm^{-1} ((2D band)[10,12]. Hybrid materials are supposed to exhibit new vibration modes and splitting of Raman modes of an individual layer. The same has been observed after the deconvolution of 2D peaks. A similar effect was observed in D peak intensity, which increased due to hybridization. The effect electric field was observed during Raman measurement by varying the electric field value from 0 V to 13 V in the RGO-RBNO system. When the voltage value was zero, Raman peaks were observed at 1296 cm^{-1}, 1351 cm^{-1} (B-N) vibration, 1586.7 cm^{-1} (G band), and 1614.9 cm^{-1} (D′) band. Similarly, when the voltage was changed to 5 V, it exhibited Raman peaks at 1314 cm^{-1} (C-N) [11] vibration mode, 1365 cm^{-1}(B-N) vibration, 1411 cm^{-1}, 1587.9 cm^{-1} (G band), and 1614.26 cm^{-1} (D′) band. Likewise, when voltage was

increased to 12 V, it displayed Raman modes at 1294.06 cm^{-1}, 1340.49 cm^{-1} (B-C)[8] vibration modes from B_4C like-structure, 1368 cm^{-1} (B-N) vibration mode, 1394.5 cm^{-1}, 1578.8 cm^{-1} (G band), and 1616.9 cm^{-1} (D′) band. When voltage was further increased to 13 V, it exhibited Raman modes at 1290 cm^{-1}, 1319 cm^{-1} (C-N)[11] vibration, 1364.7 cm^{-1} (B-N) vibration, 1576.3 cm^{-1} (G band), and 1615 cm^{-1} is due to (D′) band. Inter-layer coupling and bond formation in the hybridized (RGO-RBNO) sample, influenced by an electric field and these vibration modes, were activated when we increased the voltage[12-13]. When Raman measurement was performed under an applied electric field, the G band (C-C bond stretching mode) in RGO due to sp^2 carbon atoms was red-shifted as we increased the voltage from 0 V to 13V. Such red sifts in the G band signified electric field-induced doping in RGO-RBNO. Also, it suggests a strong modification of DOS with new states formation along with bandgap opening in the E-K energy band diagram. Also, linear E-K behavior changed parabolic nature at Γ point upon field doping.

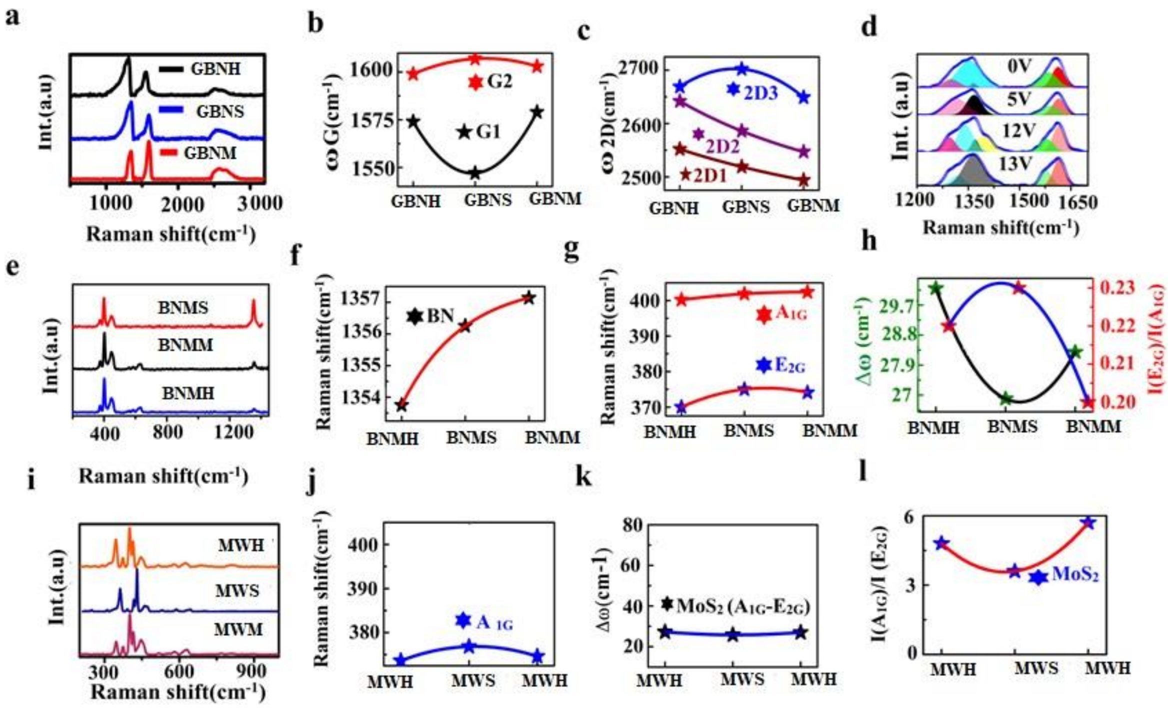

Fig. S5 (a) Raman spectra of BNH, GBNS, and GBNM hybridized samples (b-c) G and 2D peaks deconvolutions for GBNH, GBNS, and GBNM hybrids. (d) Electric field mediated Raman spectra for different voltages. (e) Raman spectra for MoS_2 and B.N. hybridized (MBNN, MBNMI, MBNH) samples, (f) blue Shifts in B.N. peaks position in hybridized samples. (g) E_{2G} and A_{1G} peak positions in hybridized BNMH, BNMM, and BNMS samples. (h) Δ ω between $E_{2g}$and A_{1g} modes due to hybridization. The minimum was observed for the MBNMI sample. (i) Raman spectra for MoS_2 and WS_2 hybridized (MWH, MWS, and MWM) samples (j) A_{1g} vibrational position of MoS_2 (k) Δ ω difference between E_{2g} and A_{1g} modes for

MoS_2 due to hybridization. (l) Intensity ratio for A_{1g}/E_{2g} peaks for MoS_2 samples when hybridized with WS_2.

Similarly, RBNO was hybridized with MoS_2 with an adequate supply of energies (temperature, pressure, and electric field energy). These layers (RBNO and MoS2) are expected to come close to each other as an effect of Mo-N, Mo-B, and N-S bond formation is desired. When hybridized sample BNMH was diagnosed with FTIR measurement, we observed differentpeaksat759,1160,961.36,1322.9,2355.1, and2330cm^{-1}. Similarly, for BNMS sample, it exhibited FTIR peaks at 768, 1375.5, 1166, 962.2, 2338, 2545.3, 2527.4, and 2603cm^{-1}. Likewise, the BNMM system displayed FTIR peaks at 761, 952.4, 1169.8, 1329.2, and 2352cm^{-1}. We have also performed the FTIR measurements for 2D B.N. exfoliated sheets to check shifts in vibration modes compared to hybridized samples. 2D B.N. sheet exhibited FTIR peaks at 758 cm^{-1} due to B-N-B out-of-plane bending mode and 946, 1327cm^{-1} due to B-N stretching mode. The out-of-plane vibration mode of B-N blue-shifted 10 cm^{-1} in BNMM hybridized sample, primarily due to interaction with neighboring MoS_2 sheets; and possibly out- of-plane bond formation. Their elastic behavior will change depending upon bonding nature (strong or weak); consequently, effective frequencies will shift from their original positions. Similar behavior was exhibited by the BNMS sample, where the B-N stretching mode got shifted by 8 cm^{-1}. Active sites available during the hybridization (B and N) are more likely to interact with MoS_2; due to the offset of S atoms from MoS_2 atomic sheets, the stretching vibration frequency of B-N will change.

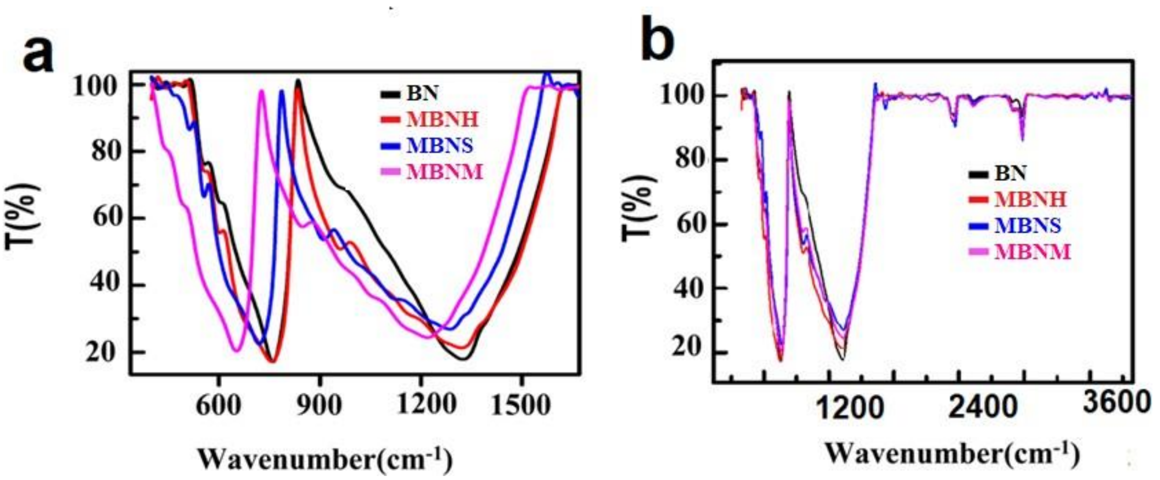

Fig. S6 FTIR spectra for B.N. and its hybrids with MoS_2(BNMH, BNMS, BNMM) (a) for a short scan range of 300 to 1800 cm^{-1}. (b) For full scan range for the hybridized (BNMH, BNMS, BNMM) samples.

When Raman measurement was performed on BNMH, it exhibited Raman peaks at 365 cm^{-1} due to hybridized (Mo-B) bond, 370 cm^{-1} (E_{2g}mode), 400 cm^{-1} (A_{1g}mode), and 449 cm^{-1} (2LA mode) modes of MoS_2 and 1357 cm^{-1} are due to B-N. It is noteworthy that a systematic blue Shift was observed in B-N (1350 cm^{-1}) vibrational mode in Raman measurements. The shifts were 3.7 cm^{-1} for BNMH, 6.2 cm^{-1} for BNMS, and 7.1 cm^{-1} for BNMM samples. Similarly, difference $\Delta\omega$ (E_{2g}-A_{1g}) modes of MoS_2 due to hybridization were found at 30.2 cm^{-1} for BNMH, 26.8 cm^{-1} for BNMS, and 28.2 cm^{-1} for BNMM samples. Decreased $\Delta\omega$ manifest hybridization and various bond formations (Mo-B and Mo-N). Also, we have found that phase transformation of MoS2 (2H to 1T) is more prominent in solvothermally hybridized samples compared to heat-mediated and microwave-based hybridized samples. We have also performed new Raman measurement in our hybrid samples and found that more prominent 1T phase Raman vibration modes **[36-37]** at ~219 cm^{-1} and ~315 cm^{-1} for Solvothermally hybridized sample (BNMS) in contrast to heat mediated (BNMH) and microwave hybridized (BNMM) samples.

When MoS_2 is hybridized with $WS_{2,}$ upon hybridization, from $MoS_{2,}$ Mo and S atom will interact with WS_2 (both active sites W and S) and vice versa; as an effect, $Mo_{1-x}W_xS_2$ like- new crystal structure formation is expected. Raman vibration mode E_{2g} (in-plane vibration) of constituent atoms, whereas A1g (out-of-plane) shift of S atom from existing (MoS_2 and WS_2) atomic sheets are sensitive to measurements. When Raman measurement was performed in hybridized MWH (heating) sample, it exhibited a peak at 288, 319, 344, 373, 400, 415, 444, 516, 576, and 625 cm^{-1}. Likewise, MWS (Solvothermal) sample exhibited Raman modes at 293, 338, 346, 375, 400, 416, 446, 577, 519 and 633 cm^{-1}. Similarly, MoS_2 was hybridized with WS_2 by microwave (MWM), and the response will differ due to their different dielectric constants in the GHz frequency range. Also, a high electric field (~10^6 V/cm) and localized heating (~ 2000 ºC). Although hybridization was performed in the liquid medium, it will push atomic sheets (MoS_2 and WS_2) even closer to each other. As an outcome, the inter-layer coupling will enhance. When Raman measurement was performed it revealed various vibration modes at 286, 322, 345, 373.5,400,414,444,516,577and622cm^{-1}. Generally, exfoliated MoS_2 sheets exhibits Raman modes at370cm^{-1} (E_{2g}), 400cm^{-1}(A_{1g}), 452cm^{-1} (2LA), 517cm^{-1} (E_{2g}+2LA), 577cm^{-1} (E'_{2g}+LA) and 622 cm^{-1} (A_{1g}+LA). Similarly, exfoliated WS_2 sheets exhibit Raman modes at 175 cm^{-1} (LA),265cm^{-1} (2LA-3E_{2G}),230cm^{-1} (A_{1g}-LA),296cm^{-1} (2LA-2E_{2g}),352cm^{-1} (2LA),355(E'_{2g}), 418 cm^{-1} (A_{1g}), 581 cm^{-1} (A_{1g}+LA) and 702 cm^{-1} (4LA). In general, Raman E_{2g} (in-plane vibration) and A_{1g} (out-of-plane vibration) modes are considered the most important Raman signature in exfoliated MoS_2 and WS_2 sheets and therefore were diagnosed minutely in hybridized sheets, and their effect on hybridization was studied**[4,18-23]**. The difference (E_{2g}-A_{1g}) in Raman modes, i.e., $\Delta\omega$ for $MoS_{2,}$ was 27.24 cm^{-1} for MWH and 25.8 cm^{-1} for MWS hybridized sample. Likewise, forWS_2difference(E_{2g}-A_{1g}) $\Delta\omega$wasfoundtobe70.54 cm^{-1} for MWHand68.9 cm^{-1} for the MWM sample. Such a significant Shift hint at the hybridization and bond formation between the MoS_2 and WS_2 system; as an effect, its intensity ratio (A_{1g}/E_{2g}) was changed.

Section 3: TEM analysis of RBNO-MoS_2 and MoS_2-WS_2 system

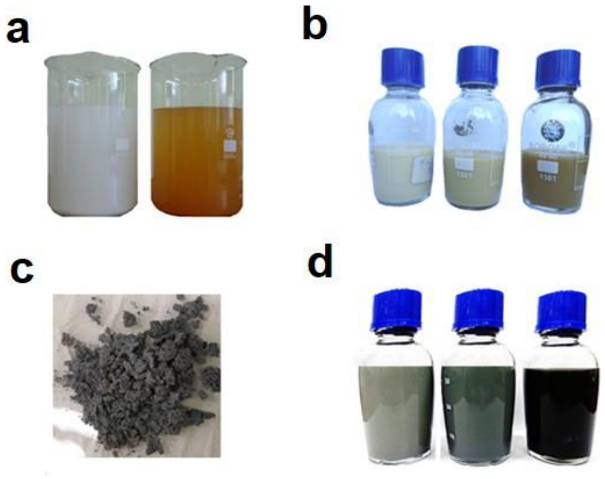

Fig. S7 Camera image of (a) synthesized BNO and G.O. sample on a large scale. (b) Mixed G.O. and BNO for hybridization with different wt. % (20, 50, and 80) in DMF. (c) Hybridized sample after processing by the heating method with wt. % (50:50) of RGO-RBNO (d) Microwave- hybridized RGO on RBNO with different wt. % from left to right (20 %, 50 % and 80 %).

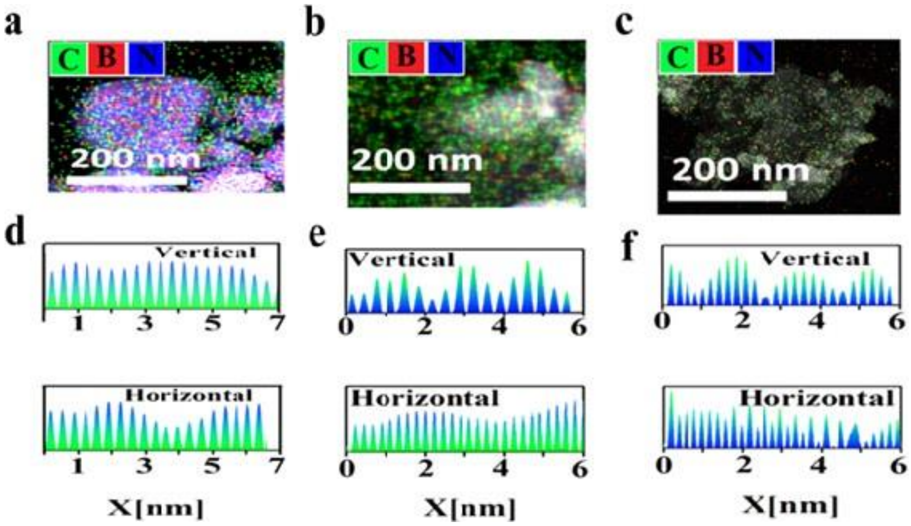

Fig. S 8 (a-c) TEM elemental mapping of GBNH, GBNS, and GBNM samples. (d-f) Atomic line profiles were obtained from HRTEM and the atomic arrays (horizontal) and across the atomic arrays (vertical) for hybridized GBNH, GBNS, and GBNM samples.

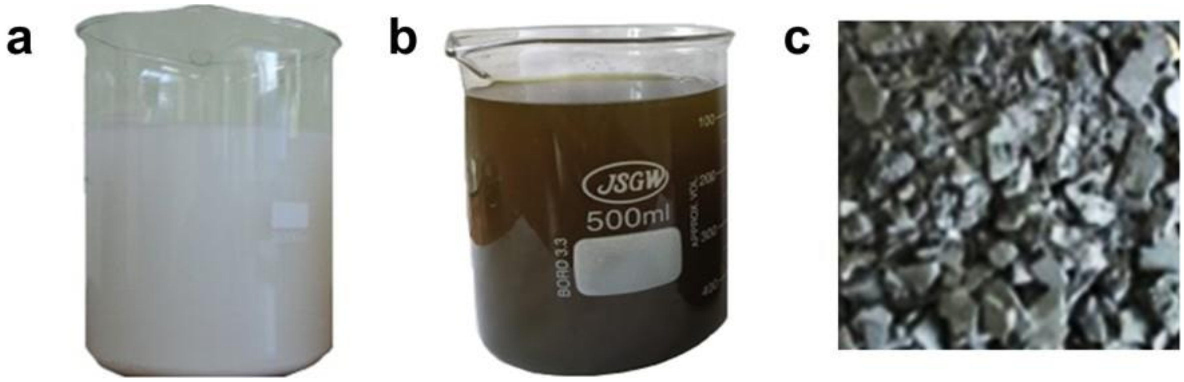

Fig. S9 (a-b) Camera image of bulk synthesis of B.N. and MoS_2 (c) Camera image of hybridized BNMH sample Wt. % (50:50).

Like the RGO-RBNO hybrid system, it is very intriguing to check hybridization and its corresponding change in the atomic arrangements of hybrid RBNO-MoS_2 systems. Therefore, we hybridized RBNO with MoS_2 by Heating, solvothermal, and microwave (see camera image in Fig. (S9)). When the BNMH sample was diagnosed with TEM (see Fig. **S10 (a)**), we observed a highly transparent sheet corresponding to the MoS_2 sheet covered with oval-shaped 2D sheets of B.N. sheets. The overlapped area was estimated at ~ 200 nm, which was further validated by elemental mapping, which gave clear proof, i.e., the presence of both MoS_2 and RBNO sheets. When the overlapped area was minutely diagnosed with HRTEM moirés pattern was observed, which signifies the hybridization between MoS_2 and RBNO (due to inter-layer coupling). When FFT was acquired in the overlapped area of HRTEM, it exhibited mixed-fold symmetry (6-fold with 2-fold) (see inset Fig. S10 (b)). Such variation in symmetry suggests the influence of foreign atomic sheets (MoS_2) over RBNO sheets. The average nuclear distance was measured in the HRTEM image across the atomic line and found to be ~2.5 Å, whereas, along the atomic line, it was ~2.1 Å. Signify the highly strained system due to strong inter-layer coupling. In contrast, the equilibrium distance for B.N. is~1.54Å, and for $MoS_{2,}$ it is~3.1 Å. Similarly, the BNMS sample was examined by TEM imaging highly transparent, and folded MoS_2 sheets were observed. This is characteristic of monolayer MoS_2 along with oval-shaped B.N. sheets. The overlap area was estimated to be ~150 nm, confirmed by elemental mapping. When the overlaying area was examined by HRTEM, we could resolve the individual atoms; the atomic arrangements also suggested a moirés pattern, possibly due to overlapping between distinct atomic sheets with some rotational angle. FFT pattern in an overlap area exhibited six-fold symmetry, possibly due to A.A. stacking. Measuring the atomic distance in the obtained moirés pattern will be interesting, as it will have different distances than their parent atomic sheets; hence we measured average atomic distances. Along the atomic line, itwas~3.45Å; across the atomic line, it was 3.0Å. It signified that MoS_2 atomic distance had

It has been reduced ~ 3 % significantly from its equilibrium distance of 3.15 Å; due to the presence of B.N. sheets in the vicinity of MoS_2 sheets.

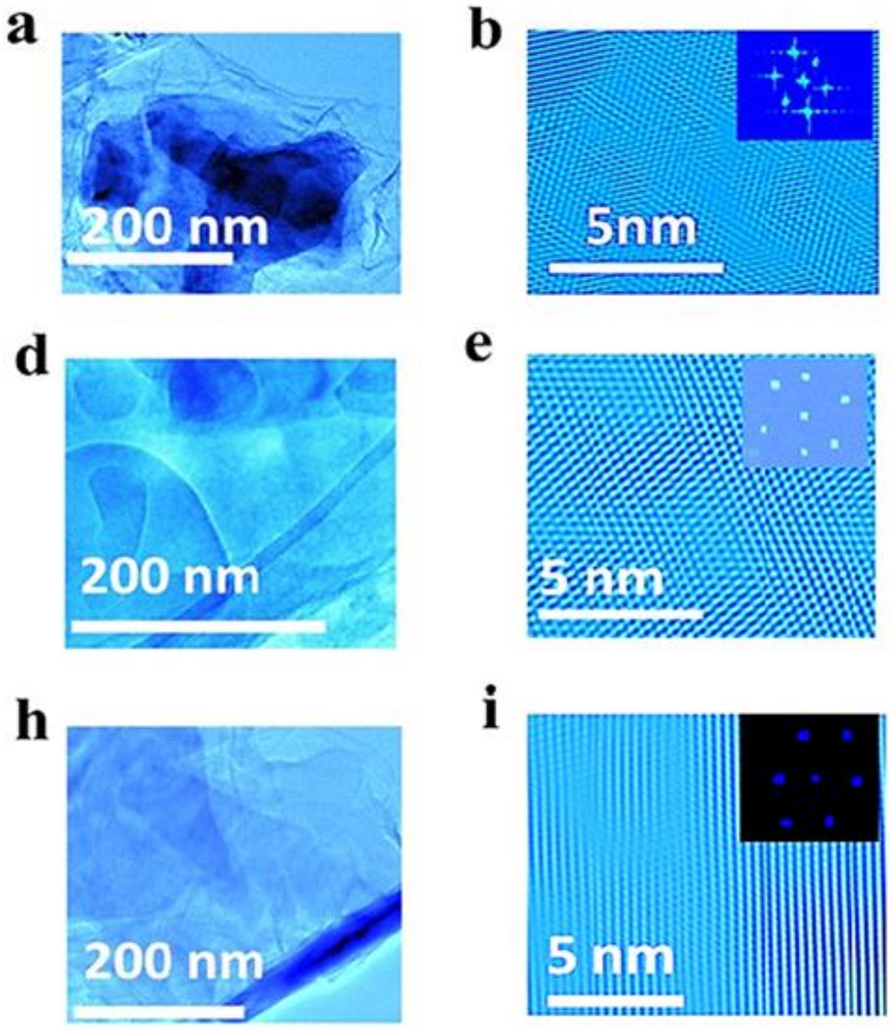

Fig. S10 (a) TEM image of RBNO-MoS_2 hybridized BNMH sample, (b) HRTEM image of the overlapped area in RBNO-MoS_2 was further analyzed by elemental mapping and in inset corresponding FFT pattern. (c)TEM image for the BNMS hybridized sample and (d) HRTEM image of an overlapped area in RBNO-MoS_2 were further analyzed by elemental mapping and, in the inset, its FFT pattern. (h)TEM image of the BNMM sample and (i) HRTEM image of the MBNM sample in the overlapped area with inset its FFTpattern.

Moreover, the MBNMI hybrid sample was hybridized in the liquid medium, where the localized temperature was moderately high with an extremely high electric field. Such dynamic conditions excelled RBNO and MoS_2 sheets close enough where bond formation occurred. When the hybridized sample was examined by TEM measurement, it exhibited transparent sheets with sharp-edged MoS_2 sheets and oval-shaped B.N. sheets extending up to more than 200 nm in lateral dimension, with an overlap area of ~100 nm. The presenceofMoS_2andRBNOintheoverlapareawasfurtherconfirmedbyelementalmapping.

The overlap area was revisited by HRTEM measurement; atoms were linearly arranged in some places (plausibly moirés pattern), whereas it exhibited hexagonal patterns in some places. When the FFT pattern was observed, it showed 6-fold symmetry. Localized change in inter-atomic distance will be exciting because it will alter its electronic and optical properties. Therefore, we carried out atomic distance measurements obtained from the HRTEM image. Along the atomic line, it was ~3.2 Å, and across the atomic arrangement, it exhibited ~3.0 Å.

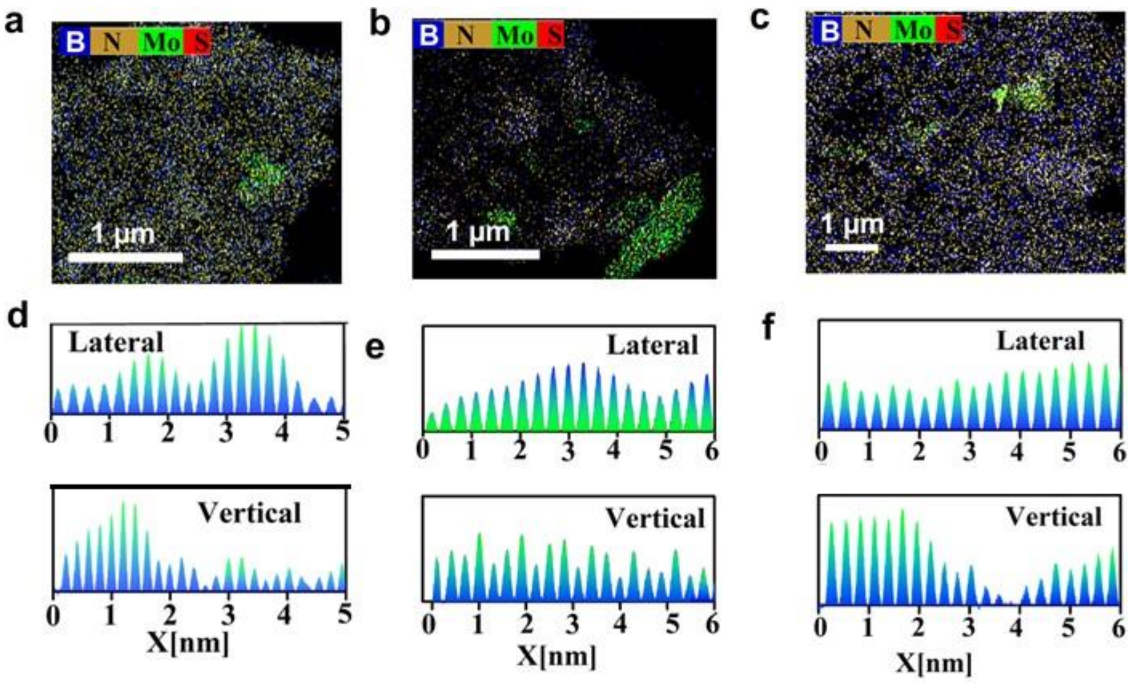

Fig. S11 Elemental mapping of RBNO-MoS_2 hybridized samples and their atomic line profiles obtained from the HRTEM images along with the atomic array (lateral) and across the atomic array (vertical). (a, d) for BNMH (b, e) for BNMS (c, f) for BNMM.

MoS_2 and WS_2 are of great interest due to their potential applications in optoelectronics, photocatalysis, sensors, and photovoltaics. Hybridizing such 2D materials will unveil various applications starting from energy storage, excitonics, transistors, and many more. Conventional hybridization techniques, e.g., CVD grow sheets, wet chemical transfer, and scotch tape transfer methods, have some limitations for device applications. The diffused interfaces during growth, residual contamination (water molecules and glue), and scalability are major concerns. We have tried to address the existing problem by exfoliating a few layers of MoS_2 and WS_2 in large quantities and hybridizing by heating, solvothermal, and microwave (see camera image of hybridized sample Fig. S12). The resulting hybridized sample was diagnosed by TEM imaging to know the effect of hybridization at the atomic scale. When the hybridized MWH sample was

Analyzed with TEM, observed highly transparent sheets with lateral dimensions of more than ~150 nm correspond to MoS_2 sheets, covering WS_2 sheets of ~30 nm (see Fig. S13 (a)). The presence of both atomic sheets in the overlap area was confirmed by elemental mapping (see Fig.S12 (d)). When overlapping areas 1& 2 were minutely diagnosed with HRTEM imaging, we noticed a hexagonal pattern in a few places along with a moirés pattern (characteristic of inter-layer coupling) that suggests interaction leading to bond formation amongst atomic sheets. The hybridized area was further studied by measuring the interatomic distance and found across the atomic line ~3.15Å and along the atomic line ~3.0 Å. Similarly, MWS hybridized sample was diagnosed by TEM imaging; we observed highly transparent folded WS_2 sheets of ~ 200 nm wrapping the MoS_2 sheet, which were tiny sheets of ~ 50 nm(See Fig.S13 (b)). Elemental mapping confirmed the coexistence of MoS_2 and WS_2 sheets. When the overlying areas 1&2 were minutely examined by HRTEM measurement, they exhibited the atomic arrangement in a hexagonal manner. The FFT pattern further validated this; it showed (6-fold symmetry). Atomic distances obtained from HRTEM along the atomic line were ~3.15Å, whereas, across the atomic line, it was ~3.0Å. When MWM hybridized sample was examined by TEM measurement, multilayer highly transparent sheets were observed lengthened up to ~250 nm, covering the WS_2 atomic sheets ~30 nm (see Fig.S13 (c)). Elemental mapping revealed the presence of both MoS_2 and WS_2 sheets. When such overlapped areas 1&2 were diagnosed with HRTEM, it exhibited a moirés pattern over the entire area; it signifies the strong inter-layer coupling as an effect of high electric field and supercritical heating under liquid medium. FFT pattern was performed over the underlying area; it exhibited six-fold symmetry, which suggests possible A.A. stacking. Inter-atomic distances were measured over the moirés pattern vertically and horizontally from the HRTEM image; it turned out that the vertical distance was ~3.15 Å and was larger than the horizontal distance of ~ 3.0Å.

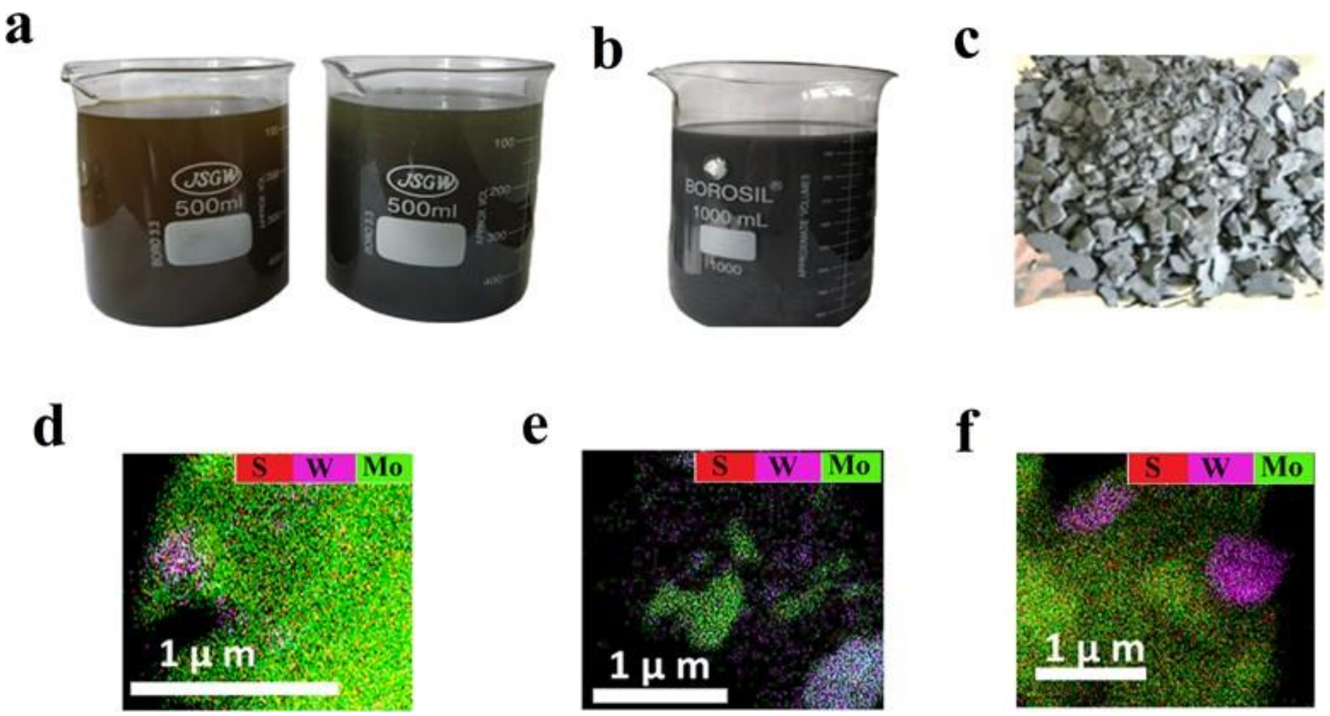

Fig. S12 (a-b) Camera image of large-scale synthesis of MoS_2 and WS_2 2D sheets and its hybrid, (c) camera image of processed MWH hybrid sample (d-f) elemental mapping of hybridized (MWH, MWS, and MWMI) samples.

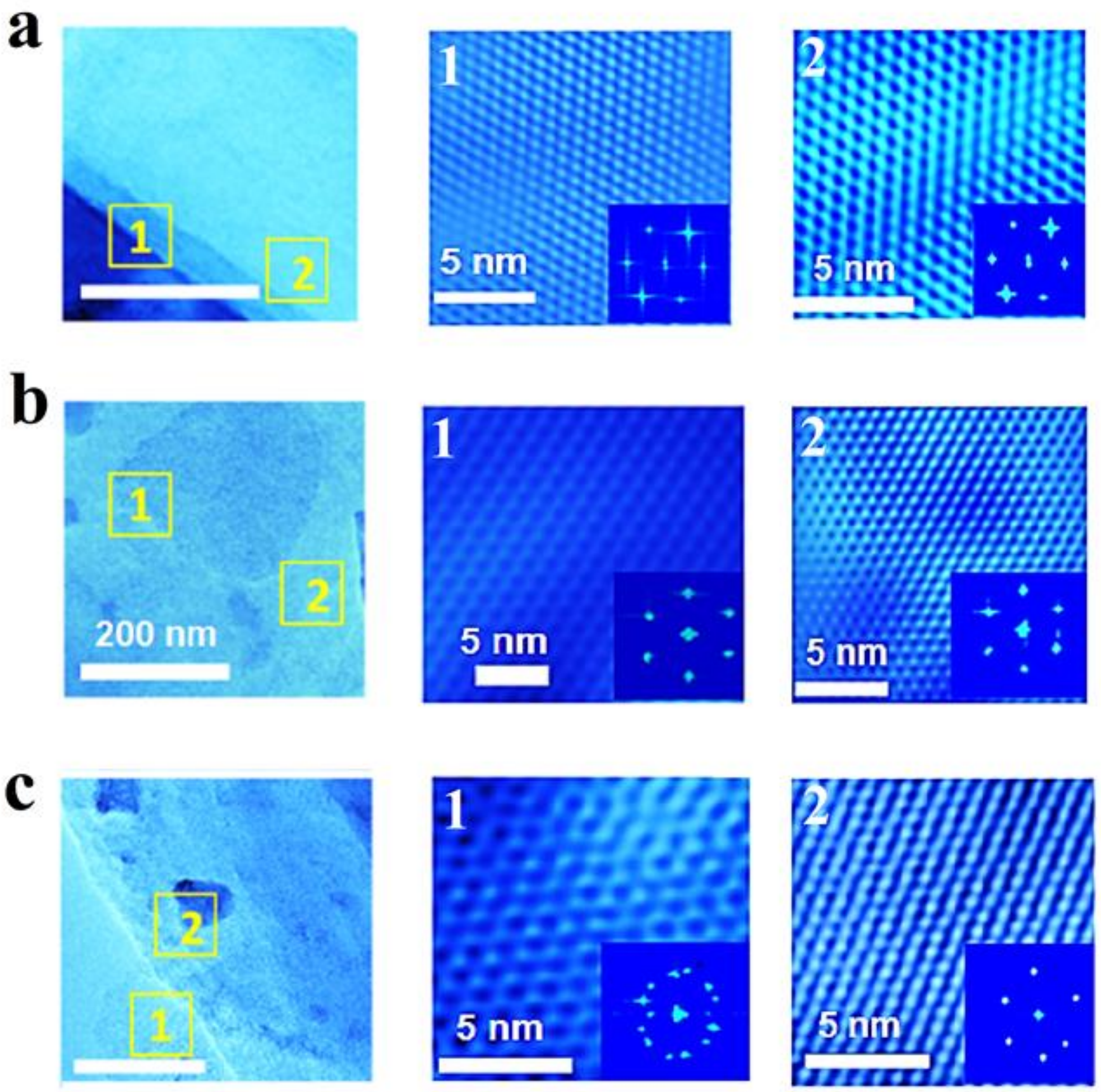

Fig S13 (a) TEM image of MWH, overlapped areas are marked as 1 and 2 and corresponding HRTEM inset their FFT. (b) TEM image of MWS, overlapped areas are marked as 1 and 2 and corresponding HRTEM inset their FFT (c) TEM image of MWM, overlapped areas are marked as 1 and 2 and corresponding HRTEM inset their FFT.

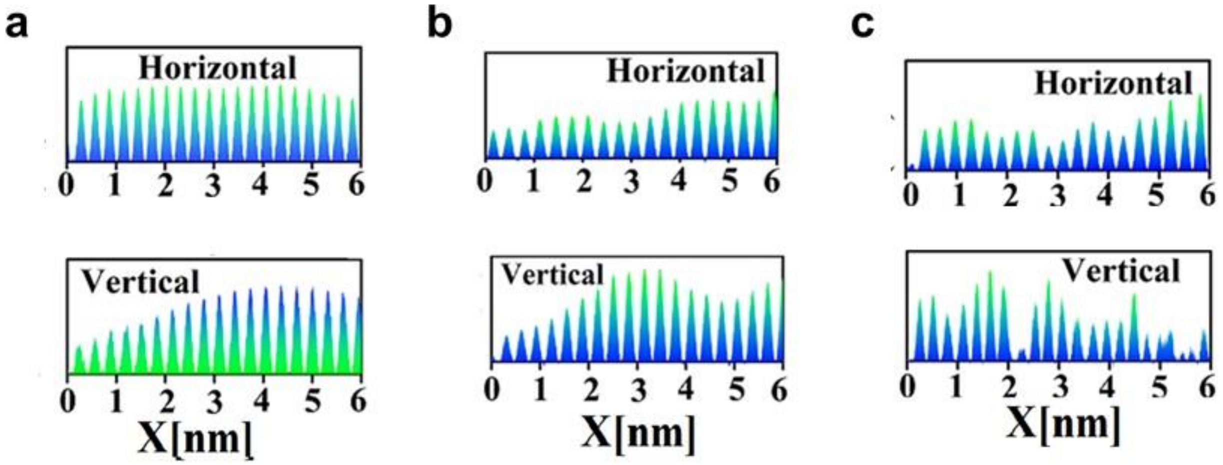

Fig. S14 Atomic line profile obtained from HRTEM images for a hybridized sample along with the atomic array (horizontal) and across the atomic array (vertical) for (a) MWH, (b) MWS, and (c) MWM samples.

Section 4: XPS details of RGO-RBNO RBNO-MOS_2 and MoS_2-WS_2 hybridized sample

Effect of hybridization on hybridized samples (GBNH, GBNH, and GBNM) attained by various processing conditions (heating, solvothermal, and microwave) and their effect on inter-layer distance and the interlayer coupling in-depth. Due to dynamic processing conditions is very important to diagnose with XPS for better understanding. XPS measurements were performed on hybridized GBNH sample (Survey shown in Fig.S15 (a)) and a short-range scan for B*1s*, C*1s,* and N*1s* spectra (see Fig.S15(b)-(c)). Upon deconvolution of B*1s,* spectra's stretches at189.6eV(B-C), and 190.9 eV (B-N) bonds were obtained. Similarly, after deconvolution of C1s spectrum, we observed stretches at 282.8eV(B-C) B_4C like-structure,283.4eV(B-C) BC_3 like-structure, 284.2 eV(C-C) bond, 285.5 eV(C-N) bond, and 288.0 eV corresponding to C-O bond. Likewise, the N1s spectrum after deconvolution exhibited stretches at 397.1eVcorresponding to the B-N bond,98.4 eV C-N pyridinic like a bond, and 399.3 eV (C=N) pyrrolic like a bond. Inter-layer coupling was expected to get even stronger when additional dynamic condition pressure was involved in hybridization. Therefore, we carried out an XPS measurement of the GBNS sample (survey shown in Fig.S15 (d)) and a short-range scan for B*1s,* C*1s,* and N*1s* spectra (See Fig. S15 (e)- (f)). Upon deconvolution of B*1s* spectra, stretches at 189.7 eV (B-C) due to BC_3 like-structure and 190.6 eV due to sp^2 bonded (B-N) were obtained. Likewise, after deconvolution of the C*1s* spectrum, we observed stretches at 283.5 eV (B-C) BC_3 like-structure, 284.4 eV due to sp^2 hybridized C-C bond, 285.1eV is because of C-N and 286.60 eV Corresponding to C-O bond. Similarly, the N1s spectrum after deconvolution exhibited stretches at 397.2 eV corresponding to the B-N bond, 398.0 eV C-N due to the pyridinic bond, and 405.5 eV because of the N-O bond. When microwave was employed for hybridization, dielectric heating due to oscillating electric field and high electric field will catalyze the reaction, and inter-layer distance will be further reduce. When XPS measurement was performed on GBNM hybrid sample (see survey in Fig. S15 (g)), and short-range scans for B1s, C1s, and N1s spectra (see Fig. S15 (h-i)). Upon deconvolution of B1s spectra, stretches at 189.7 eV (B-C) bond and 190.7 eV (B-N). Likewise, the N1s

spectrum after deconvolution exhibited stretches at 397.2 eV corresponding to the B-N bond, 398.0 eV(C-N) due to pyridinic-like bond, and at 405 eV (N-O) like a bond. Similarly, after deconvolution of C1s spectrum exhibited different stretches at 283.0 eV (B-C) BC_3 like-structure 284.0 eV due to sp^2 bonded C-C, 285.1 eV due to pyridinic like structure (C-N) and at 287.6 eV corresponding to N- C=O bond were obtained. Various bond % due to different techniques of hybridization were estimated and it was found that the B-C bond was 31 % for GBNH, 37.5 % for GBNS, and 5 % for GBNM hybridized sample. Likewise, the same calculation was performed for the C-N bond, and it was found to be 24 % for GBNH, 27.1% for GBNS, and 50.2% for the GBNM sample. We also observed that B-N bonding % decreased from 27 % for GBNH to 23 % for GBNM hybridized sample because B and N participated in bond formation with C atoms[11,14,15-16].

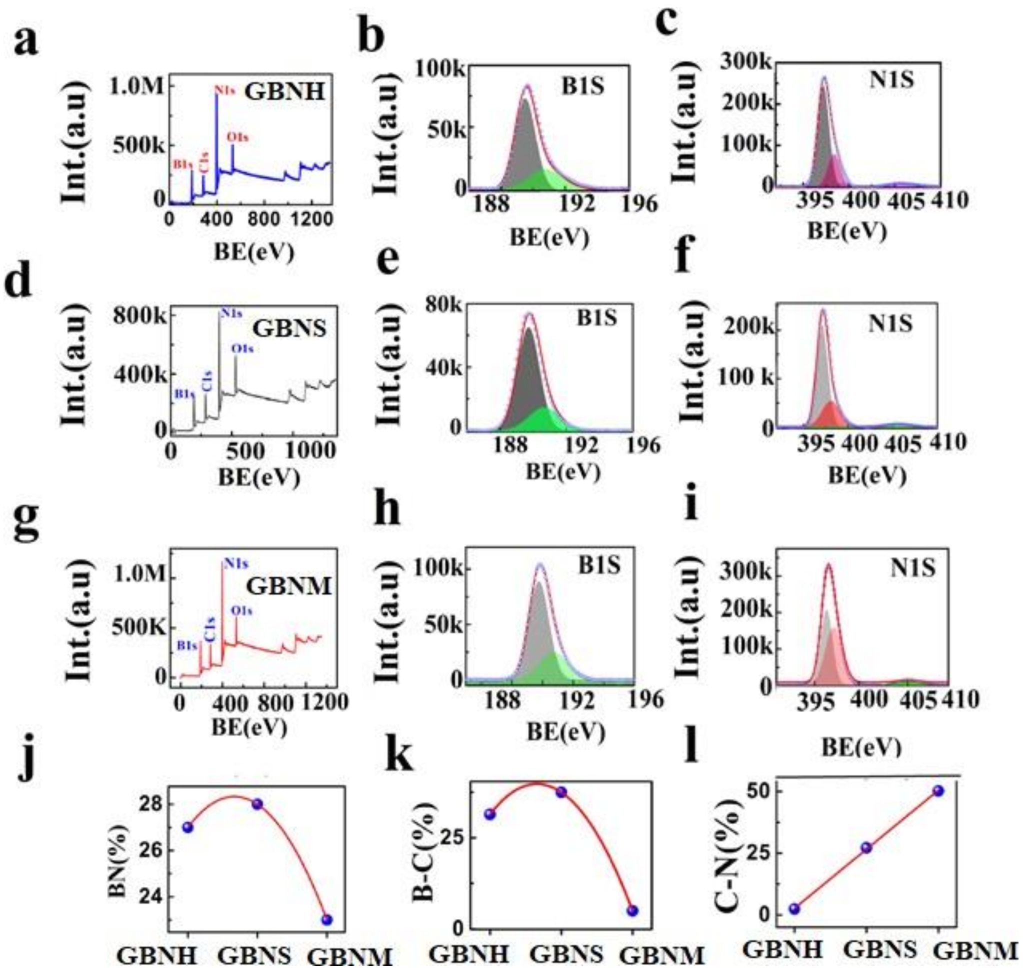

Fig. S15 XPS spectrum for RGO-RBNO hybridized samples full range scan (a) for GBNH, (d) for GBNS, and (g) for GBNM hybrid samples. (b-c) short scan range for B 1s and N 1s spectra for GBNH. (e-f) short-range scans (180 to 196 eV) for B1s and N 1s (390 to 410 eV) spectra for the GBNS sample. (h-i) short-range scans (180 to 196 eV) for B1s and N 1s (390 to 410 eV) spectra for the GBNM sample. (j) B-N bonding % estimated for GBNH, GBNS, and GBNM obtained from B 1s, and N 1s spectra, and (k) B-C bonding % calculated for 1 GBNH, 2 GBNS, and 3 GBNM obtained from B1s and C1s spectrum and (l) C-N bonding % GBNH, GBNS, and GBNM derived from C1s and N1s spectra. Similarly, the hybridized BNMH sample was diagnosed by XPS measurements (survey showed in Fig.S16 (k)) and short-range scan for B 1s, Mo 3d, N 1s, and S 2p spectra (See Fig. S16 (l)- (o)). Upon deconvolution of the B1s spectrum, stretches at 189.2 eV, 189.8 eV owing to Mo_3B-like structure (Mo-B), and at 191.0 eV assigned to sp^2 structure of B.N. (B-N) were obtained. Likewise, N1s spectrum after deconvolution displayed stretches at 394.0 eV corresponding to Mo-N bond 397.1eV, 398.2 eV due to B-N, and 405.3 eV because of N-O bond. Likewise, Mo 3d spectrum exhibited stretches at 225.3 eV ascribed to Sulphur 2s, 227.5 eV, and 228.1 eV due to the Mo-B bond

attributed to MoB_2 like-structure, 228.7 eV ($3d_{5/2}$) and 231.2 eV($3d_{3/2}$) of metallic 1T phase of MoS_2, 231.7 eV (3d $_{5/2}$) ascribed to semiconductor 2H phase of MoS_2. Similarly, after the S 2P peak deconvolution, it exhibited stretches at 161.0 eV ($2P_{3/2}$) and 162.25 eV ($2P_{1/2}$) owing to the metallic 1T phase of MoS_2, 163.6 eV, and 167.8 eV is because of elemental Speak. Similarly, the BNMS hybrid was examined by XPS (survey shown in Fig.S16 (a)) and short-range scan for B1s, Mo 3d, N1s, and S2p spectrum (See Fig. (b)- (e)). Upon deconvolution of B1s spectra, stretches at 189.2 eV and 189.4 eV owing to Mo_3B -like- structure (Mo-B) bond and 191.1 eV assigned to sp^2 like structure of B.N. (B-N) bond and 192.2eV due to (N-O) bond. Likewise, N1s spectrawerede convolutedit displayedstretchesat394.1eV corresponding to (Mo-N) bond 397.0 eV, 398.2 eV due to (B-N), and 405.3 eV because of the N-O bond. Likewise, Mo 3d spectrum exhibited stretches at 225.4 eV assigned to sulfur 2s, 227.6 eV and 228.2 eV due to (Mo-B) bond attributed to MoB_2 like- structure, 228.7 eV ($3d_{5/2}$) and 231.2 eV($3d_{3/2}$) ascribed of metallic 1T phase and at 231.8 eV($3d_{5/2}$) which signifies 2H phase of MoS_2. Similarly, after the S 2P peak deconvolution, it exhibited stretches at 160.9 eV ($2P_{3/2}$) and 162.1 eV ($2P_{1/2}$) owing to the 1T phase of MoS_2, 163.6 eV, and 167.8 eV because of elemental Speak. Hybridized sheets obtained by microwave method (BNMM sample) were scrutinized by XPS (see survey in Fig. S16 (f)), and their short-range scan for B1s, Mo 3d, N1s, and S2p spectra (See Fig. (g- j)). Upon deconvolution of B1s spectrum, stretches at 189.9 eV due to Mo_3B like-structure Mo-B bondand191.1 eV ascribed to sp^2 like the structure of a B.N. (B-N) bond. Likewise, for the N1s spectrum after deconvolution, it exhibited stretches at 394.3eVcorrespondingtoMo-Nbond,397.4eV,398.7 eV due to B-N, and 406.6 eV because of the N-O bond. Likewise, Mo 3d spectra displayed stretches at 225.7 eV ascribed to sulfur 2s, 228.2 eV (Mo-B) bond attributed to MoB_2 like- structure, 228.7 eV ($3d_{5/2}$) and 231.2 eV ($3d_{3/2}$) signifies 1T phase of MoS_2, 232.7 eV (3d $_{5/2}$) ascribed 2H phase of MoS_2. Similarly, after deconvolution of the S 2*P* peak, it exhibited stretches at161.2 eV ($2p_{3/2}$) and 162.8 eV ($2p_{1/2}$) owing to the metallic 1T phase of MoS_2, at 162.4 eV ($2p_{3/2}$) due to 2H phase of MoS_2 and at 167.8 eV is because of elemental S peak.

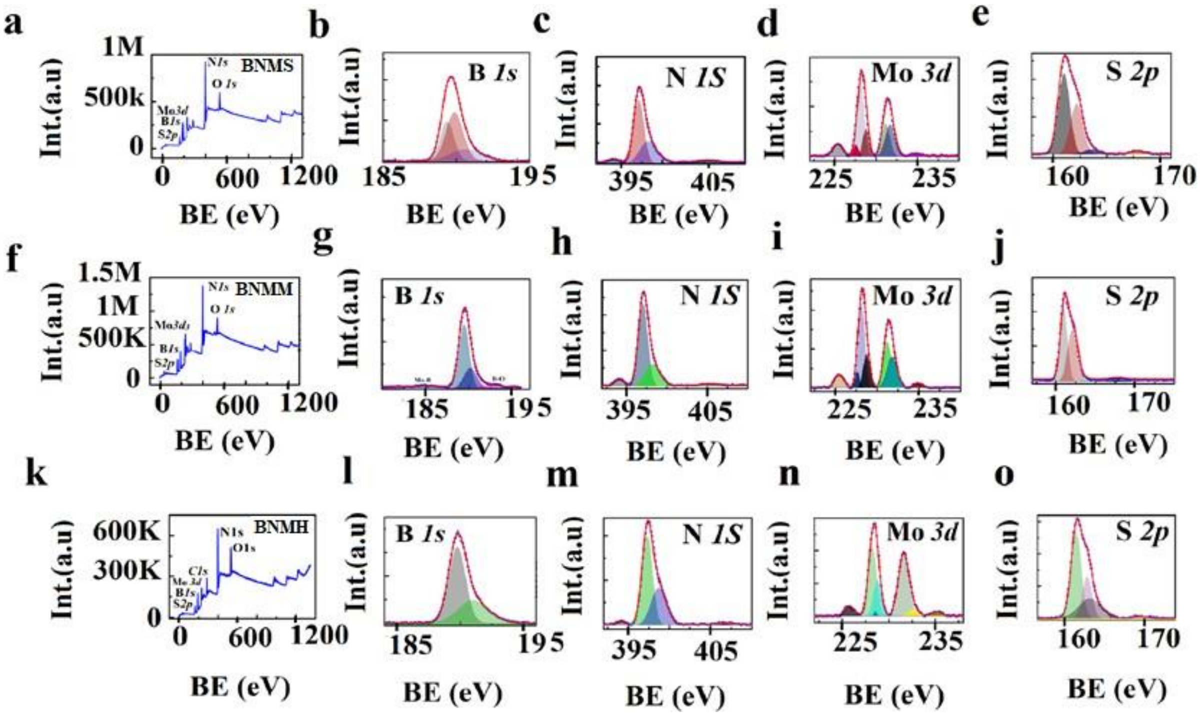

Fig.S16XPS spectrum for (MoS_2-RBNO) hybridized samples full range scan(0-1400eV) for (a) BNMS, (f) BNMM, and (k) BNMH (b-e) short-range scans for B1s, N 1s, Mo 3d, and S2p for BNMS sample(g-j) short-range scans forB1s, N1s, Mo 3d and S2p for BNMM sample and (l-o) short-range scan for B1s, N1s, Mo 3d and S 2p for BNMH sample.

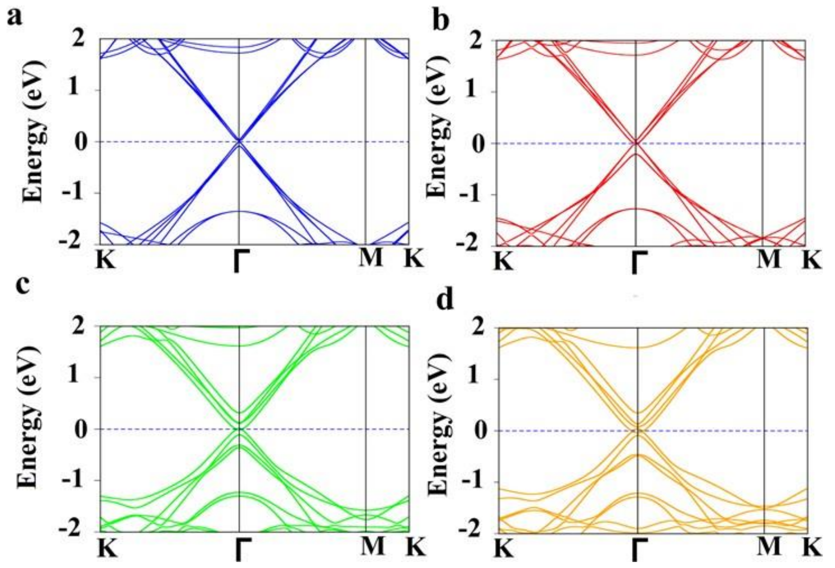

Fig. S17 (a-d) DFT band structure calculations for Gr-BN hybridized samples for A.A. stacking sequence for two distances 4.5 and 3 Å.

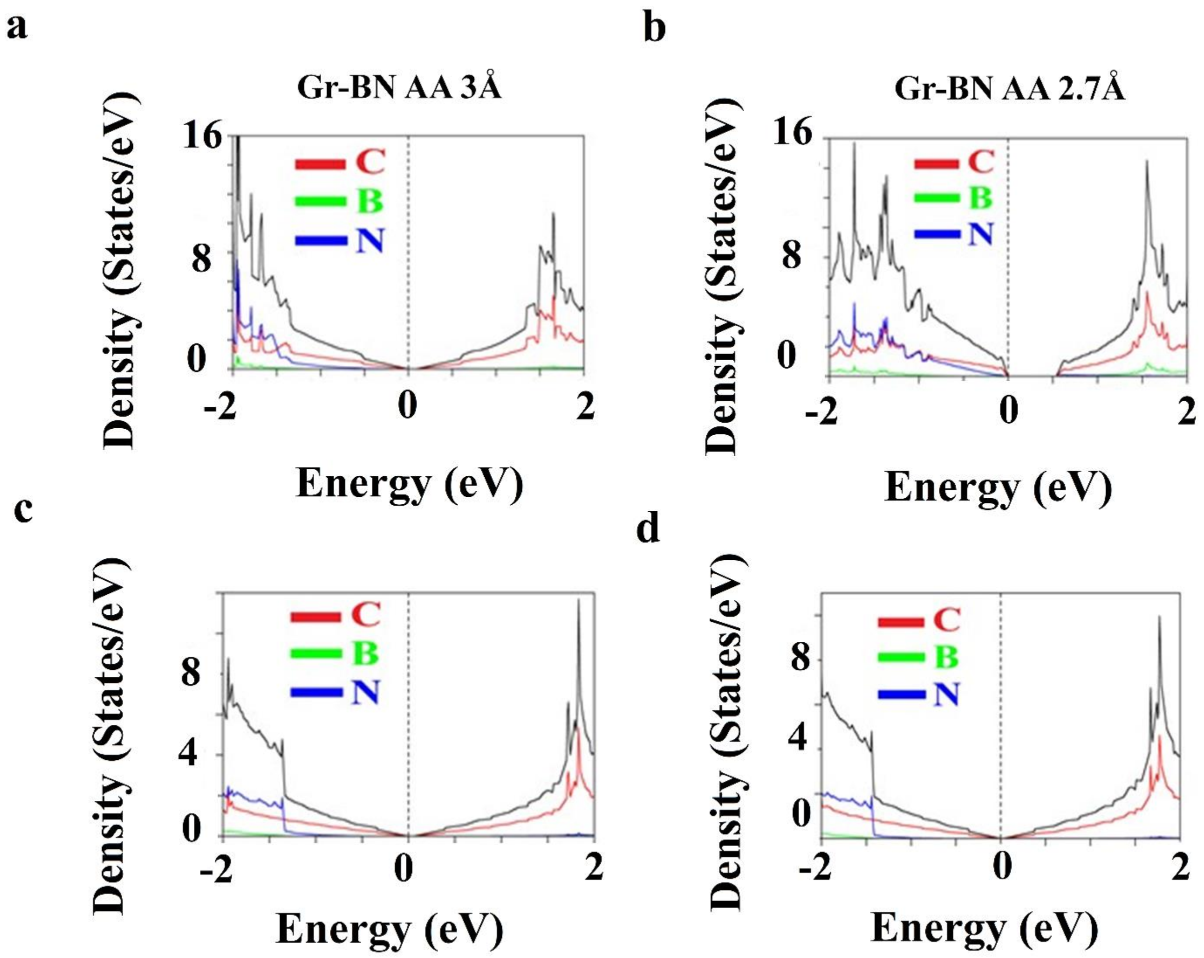

Fig. S18 Density of state for Gr-BN hybridized samples for A.A. stacking for two distances (a) 3.0 Å (b) 2.7 Å and Gr-BN AB stacking sequence with inter-layer distances (c) 3 Å and (d) 2.7 Å.

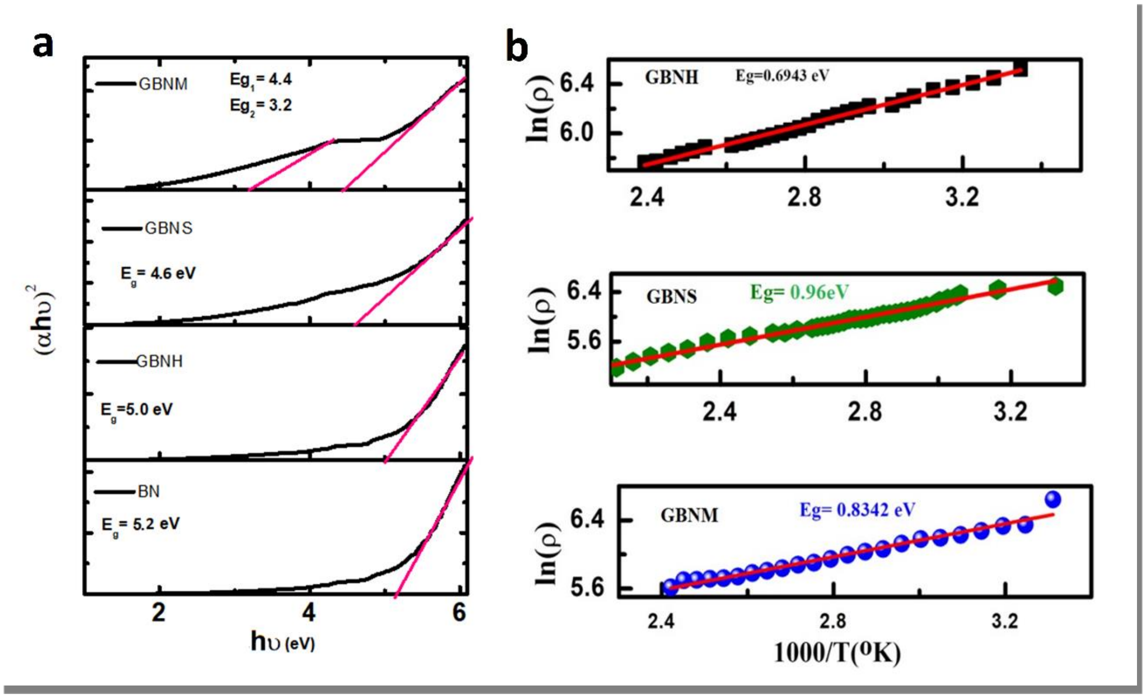

Fig. S19 (a) Tauc plot for GBNH, GBNS, GBNM, (b) Logarithmic resistivity vs. (1000/temperature) graph for RGO-RBNO hybrid (GBNH, GBNS, and GBNM) samples.

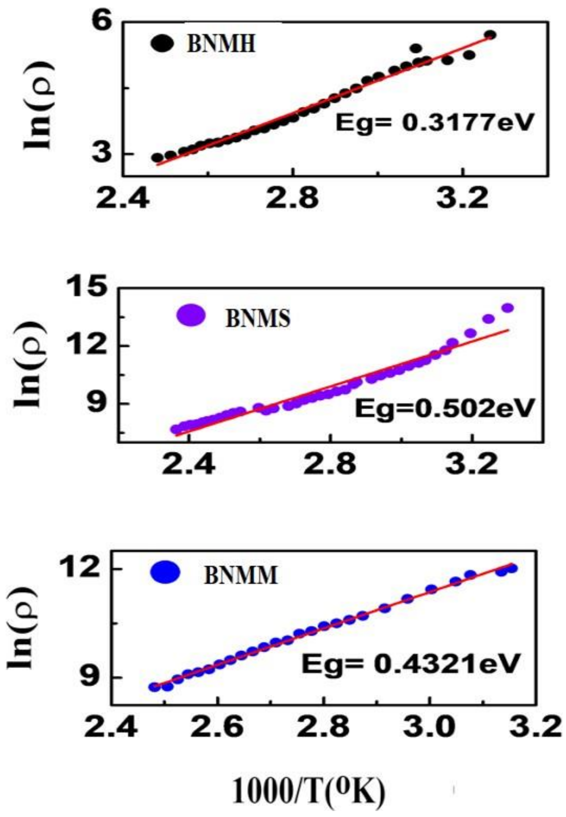

Fig. S20 Resistance Vs. (1000/temperature) graph for MoS_2-RBNO hybridized (BNMH, BNMS, and BNMM) samples.

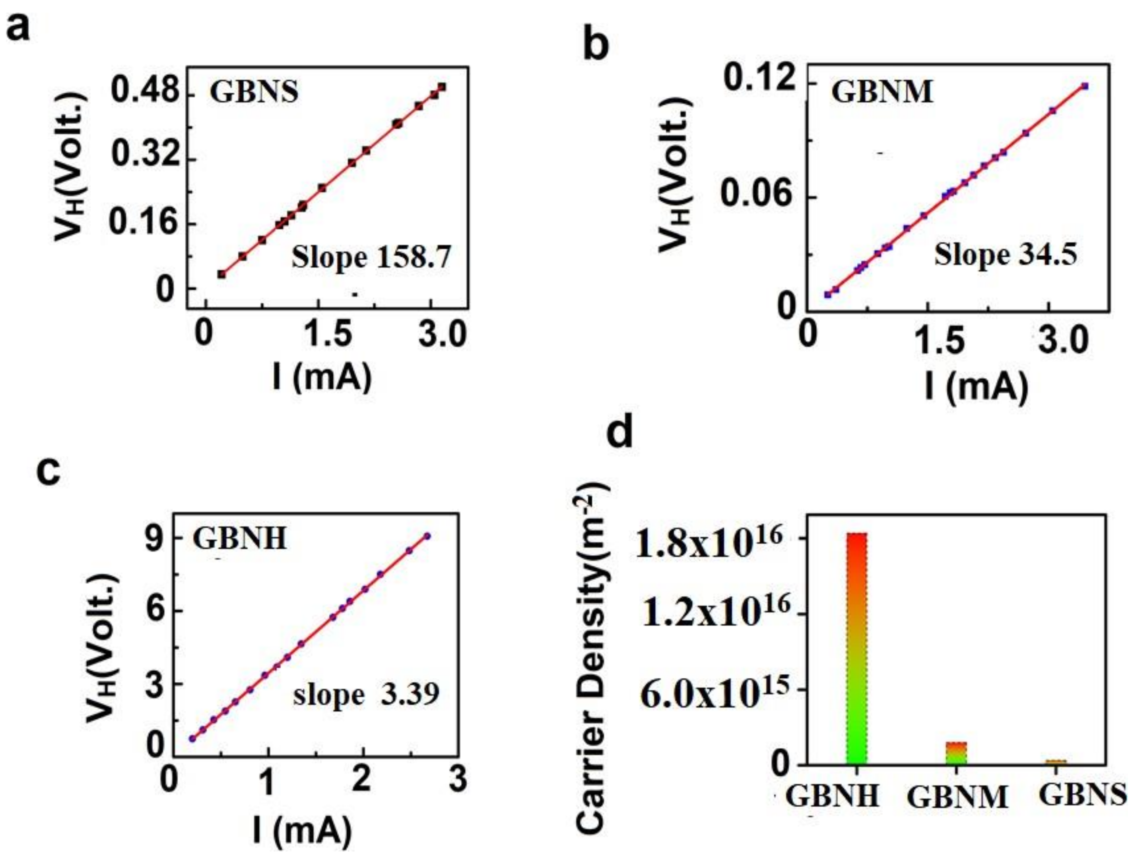

Fig. S21 Hall coefficient measurements for RGO-RBNO hybridized samples (a) GBNS (b) GBNM (c) for GBNH (d) Carrier density graph for GBNH (N), GBNS (S), and GBNM (Mi)**.**

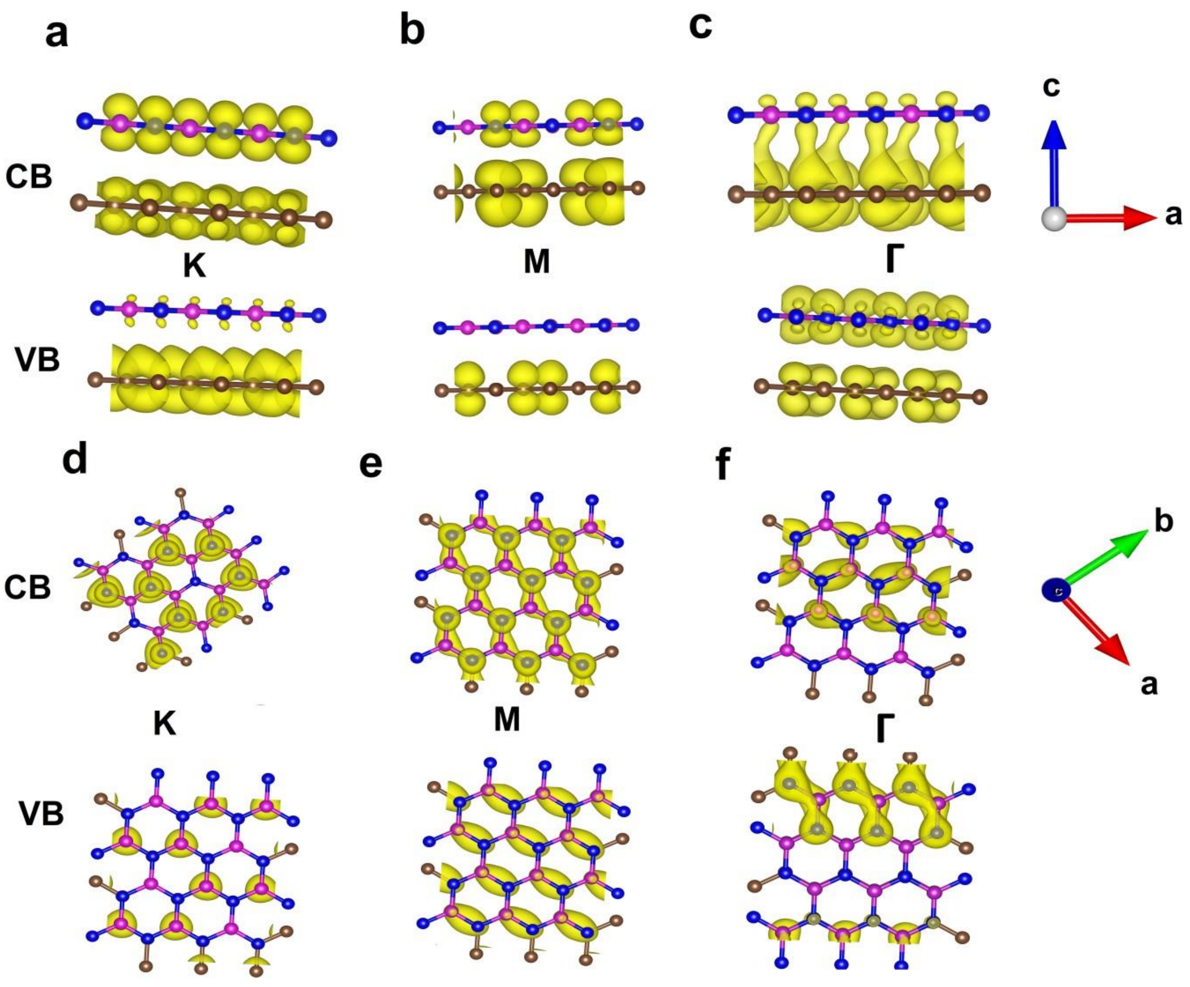

Fig. S22 Charge density difference profiles for conduction band and valance band when the inter-layer distance was 2.7Å for Gr-BN system at different dispersion (K, M, and Γ) points (a-c) Side view and (d-f) top view.

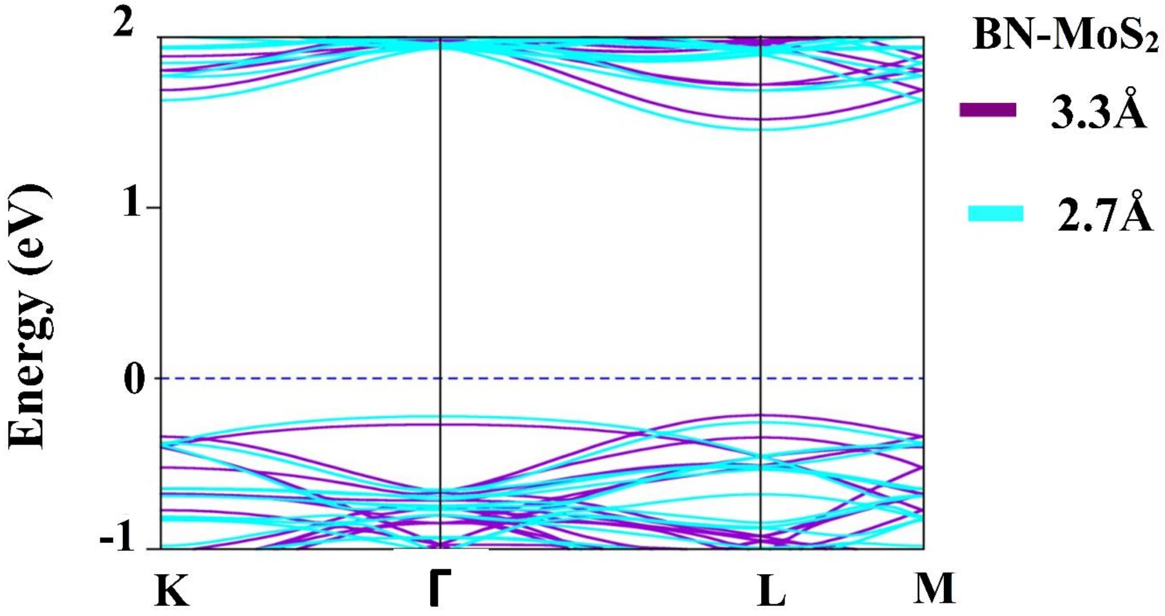

Fig. S23 DFT band structure calculation for BN-MoS_2 hybrid when the inter-layer distance was reduced from 3.3 Å to 2.7 Å, exhibited band gap was increased from 2.7 eV to 2.9 eV.

Section 5: Bond formation energies of various bonds and suitability of the techniques

In our hybridization techniques, such as the solvothermal technique, pressure is the main candidate that decides the chemical bond formation between the different atomic sheets. The pressure built up inside the solvothermal reactor depends upon the choice of solvents and temperature of the reactor. In our case, we've chosen DMF due to its reducing nature, and the reactor temperature was ~200℃. The experimentally available DMF pressure is ~18.806 kPa for 96 ℃ [26]. Further pressure can be estimated by using Vangner type vapor pressure equation developed for DMF solvent

$$\boldsymbol{ln}\left(\frac{p}{p_c}\right) = \frac{T_c}{T}\left(a_1\tau + a_2\tau^{1.25} + a_3\tau^3 + a_4\tau^7\right) \text{..................} \ \mathbf{(1)}$$

Where $\boldsymbol{\tau = 1 - \frac{T}{T_c}}$, a_1=36.48688, a2=-68.67427, a3=62.2708, a4 = - 229.0057. Tc = 596.6 K is the critical temperature, and pc = 5.220 MPa is the critical pressure [26].

Using the above equation for T =200℃ to the obtained vapor pressure of DMF within the reactor by substituting the constant values in the equation we obtained a pressure

is ~0.5 MPa. These conditions (pressure ~0.5 MPa and temperature~200 ℃) are sufficient for bond formation. As it was discovered in the case of graphite $sp^{2,}$ that gets converted to graphite-like Dimond (sp^2+sp^3) at elevated temperature and pressure. The P-T diagram for such conversion is shown in Fig.S24 [27]

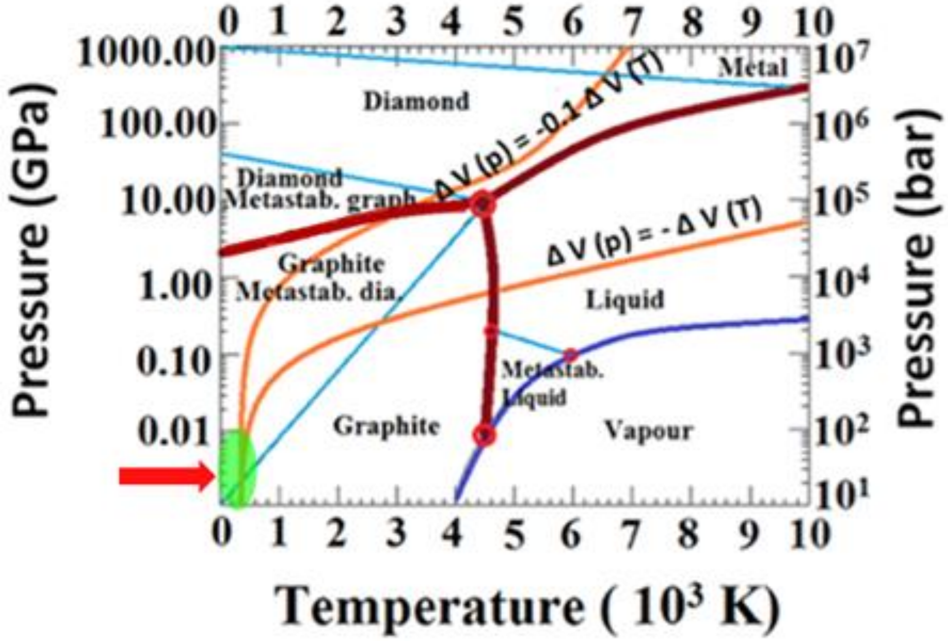

Fig.S24. P-T phase diagram of carbon the light green color represents our experimentally obtained temperature and pressure zone, where the chemical bond formation occurs.

Similarly, the microwave-based processing method for hybridizing different atomic sheets depend upon microwave power. The microwave power absorption relies upon the material's dielectric and magnetic properties (if the sample is magnetic in nature). The power absorbed by the material inside the microwave reactor in liquid medium is given by

$$\boldsymbol{P = 2\pi f(\epsilon_0 \varepsilon' E^2 + \mu_0 \mu' H^2)} \text{.................................... } \textbf{(2)}$$

Where P is power f is the frequency of the microwave, E is the electric field because of microwave, ϵ_0= 8.8X10-14, ε'represent the relative dielectric constant due to material and solvent, μ_0 =4π × 10−7 H/m, μ' represent relative permeability of the material and H is the magnetic field. Our materials are intrinsically nonmagnetic in nature; therefore, the second term isn't significant. So, the power absorbed by the material is given by

$$\boldsymbol{P = 2\pi f\left(\epsilon_0 \varepsilon' E^2\right)} \dots\dots\dots\dots\dots\dots\dots\dots\dots\dots\dots\dots\dots\dots\dots\dots (\mathbf{3})$$

GBNM hybrid material dielectric constant is ε'=6.8 at 2.45 GHz obtained from experimental measurement as shown in Fig.S25 for GBNS, GBNH, and GBNM samples.

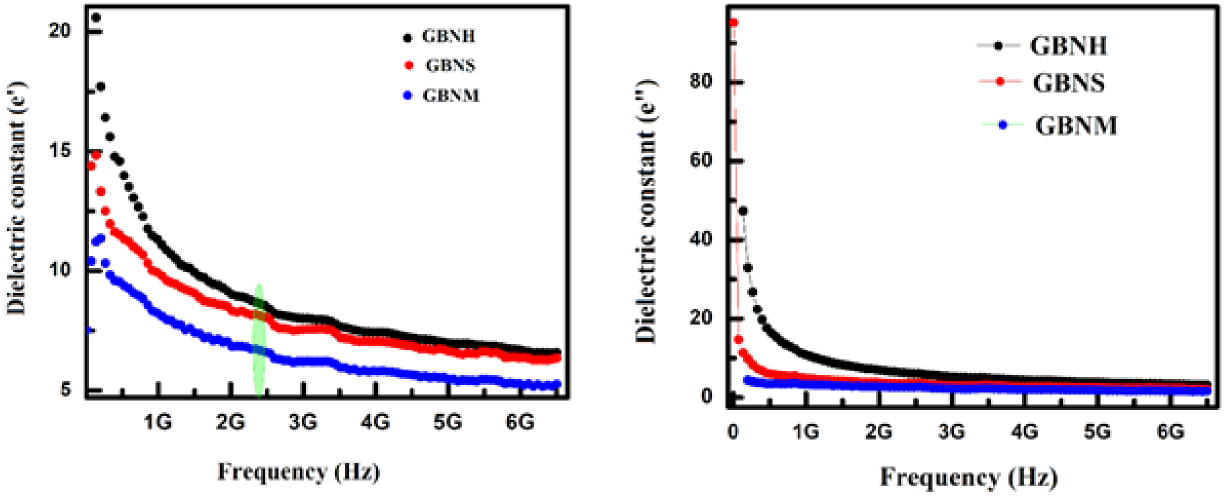

Fig. S25 Dielectric measurement for GBNS, GBNM, and GBNH samples. The light green oval shape represents the interest area.

The distribution of the electric field inside the microwave oven was obtained from the simulated result. As shown in Fig. S26 [28]

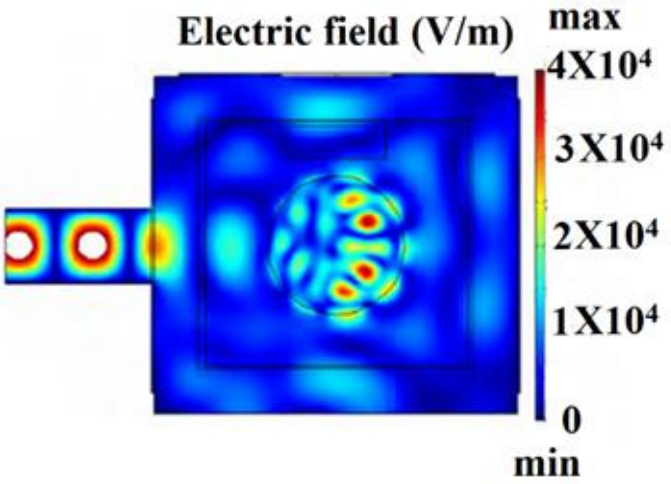

Fig. S26 Electric field distribution for the sample with ε′= 6.25 and frequency of 2.45 GHz [28].

Using dielectric constant ~ 6.8 obtained from experimental dielectric measurement for GBNM hybrid and electric field value ~2 x10^2 to 4x10^4 V/m [29-30] obtained from numerous simulation results, in equation (3) and calculated absorbed power for our materials, it turns out to be ~ 0.38 eV/atom. When the microwave is brisked in a pulsed manner of ~ 60 s, the global temperature inside the reactor where DMF is present can go up to ~200 ℃ within a very short duration of time, ~ 60s [31], which will cause additional pressure ~0.5MPa inside the microwave reactor. The energy because of pressure built up inside the reactor is ~ 0.41 eV. Hence the adequate energy received by atoms within the microwave oven is ~ 0.41+0.38= 0.79 eV/ atom.

The skin depth is obtained by using the subsequent formula [32]

$$d = \frac{3.32X10^7}{\omega\sqrt{\varepsilon_0}\varepsilon'\, tan(\frac{\varepsilon''}{\varepsilon'})} \text{..........................} \quad (4)$$

Here ω is the frequency of the microwave, ε'=6.8 is the real part, and ε"=2.55 is the imaginary part of the dielectric constant at 2.45GHz in the case of the GBNM sample. Substituting all values in equation (4) to obtain penetration depth, it turns out to be ~ d=11.47 mm. Thus, our material exhibited low loss, which is suitable for better microwave absorption and minimal conversion of microwave energy into heat energy.

The bond formation energies because of varied bonds C-N ~ 0.32 eV, B-C ~1.62 eV, [33], Graphitic N ~0.06 eV, Graphitic N2 ~ 0.173 eV [34], nitrogen and boron bonded together with carbon atoms ~ 0.168 eV, 2 nitrogen bonded with graphene ~1.69 eV and 2 boron bonded with graphene ~1.53 eV [35]. Our synthesis techniques Heat mediated, solvothermal, and microwave-based hybridization techniques offer the energies ~ 0.05 eV, 0.46 eV, and 0.916 eV/atoms that are sufficient for bond formation.

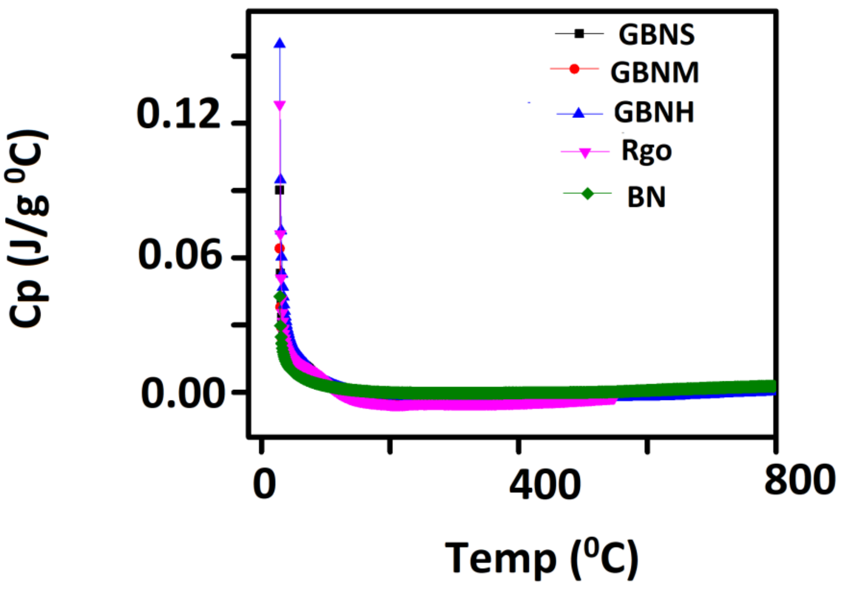

Fig. S27 Specific heat variation with temperature for RGO, BN, GBNS, **GBNH** and GBNS samples.

Reference

1. D.A. Nguyen, T. S. Le, D. Y. Park, D. Suh, and M. S. Jeong. Synthesis of $MoWS_2$ on flexible carbon-based electrodes for high-performance hydrogen evolution reaction. *ACS Appl. Mater. Interf.* **11**, 37550-37558(2019).

2. H. Ahmad, A. S. Sharbirin, and M. F. Ismail. Molybdenum tungsten disulfide ($MoWS_2$) as a saturable absorber for a passively Q-switched thulium/holmium-codoped fiber laser. *Jour. Mod. Opt.* **66**, 1163-1171(2019).

3. H. Chi, J. Liu, X. Zhang, X. Xue, D. Zhang, X. Lin, P.Huang, et al. Synergetic defects boost charge separation in C.N. for enhanced photocatalytic water splitting. *Jour. Mater. Chem. C* **8**, 9366 (2020).

4. M. Wu, J. Zhan, K. Wu, Z. Li, L.Wang, B. Geng, L. Wang, and D. Pan. Metallic 1T MoS_2 nanosheet arrays vertically grown on activated carbon fiber cloth for enhanced Li-ion storage performance. *Jour. Mater. Chem.* A **5**, 14061-14069(2017).

5. P. R. Jothi, Y. Zhang, J. P. Scheifers, H. Park, and B. P. Fokwa. Molybdenum diboride nanoparticles as a highly efficient electrocatalyst for the hydrogen evolution reaction. *Sus. Ener. Fuels* **1**, 1928-1934(2017).

6. V. Tagliazucca, M. Leoni, and C Weidenthaler. Crystal structure and microstructural changes of molybdenum nitrides traced during the catalytic reaction by in situ X-ray diffraction studies. *Phys. Chem. Chem. Phys.* **16**, 6182-6188(2014).

7. L. Shen and N. Wang. Effect of nitrogen pressure on the structure of Cr-N, Ta-N, Mo-N, and W.N. nanocrystals synthesized by arc discharge. *Jour. of Nanomater.* 1-5 (2011).

8. K. M. Reddy, P. Liu, A. Hirata, T. Fujita, and M. W. Chen. Atomic structure of amorphous shear bands in boron carbide. *Nat. Comm.* **4**, 1-5(2013).

9. B. Ozturk, A. d. L. Bugallo, E. Panaitescu, A. N. Chiaramonti, F. Liu, A. Vargas, X. Jiang, et al. Atomically thin layers of B–N–C–O with tunable composition. *Sci. Adv.* **1**, 1500094 (2015).

10. J. Guo, L. Zhang, T. Fujita, T. Goto, and M. Chen. Pressure-induced depolarization and resonance in Raman scattering of single-crystalline boron carbide. *Phys. Rev. B* **81**, 060102 (2010).

11. C. Fan, J. Miao, G. Xu, J. Liu, J. Lv, and Y. Wu. Graphitic carbon nitride nanosheets are obtained by liquid stripping as efficient photocatalysts under visible light. *RSC Adv.* **7**, 37185-37193 (2017).

12. S. Eigler, C. Dotzer, and A. Hirsch. Visualization of defect densities in reduced graphene oxide. Carbon **50**, 3666-3673(2012).

13. J. B. Wu, M.L. Lin, X. Cong, H.N. Liu, and P.H. Tan. Raman spectroscopy of graphene-based materials and its applications in related devices. *Chem. Soc. Rev.* **47**, 1822-1873 (2018).

14. S. Wang, G. Wang, T. Wu, Y. Zhang, F. Zhan, Y. Wang, J. Wang, Y. Fu, and J. Qiu. BCN nanosheets templated by gC_3N_4 for high-performance capacitive deionization. *Jour. Mater. Chem. A* **6** 14644-14650 (2018).

15. B. Gupta, N. Kumar, K. Panda, V. Kanan, S. Joshi, and I. V. Fisher. Role of oxygen functional groups in reduced graphene oxide for lubrication. *Sci. Rep.* **7**, 1-14(2017).

16. A. Prakash, S. D. Nehate, and K. B. Sundaram. Boron carbon nitride-based metal-insulator-metal UV detectors for harsh environment applications. *Opt. Lett.* **41**, 4249- 4252 (2016).

17. J. Qu, Y.Wang, J. Guo, Y. Dong, and T. Lou. Sensitive simultaneous determination of hydroquinone and catechol based on BCN graphene and poly (alizarin red S). *Jour. Electrochem. Soc.* **161**, 220(2014).

18. M. Thripuranthaka, R. V. Kashid, C. S. Rout, and D. J. Late. Temperature-dependent Raman spectroscopy of chemically derived few-layer MoS_2 and WS_2 nanosheets. *Appl. Phys. Lett.* **104**, 081911(2014).

19. W. Zhao, Z. Ghorannevis, K. K. Amara, J. R. Pang, M. Toh, X. Zhang, C. Kloc, P. H. Tan, and G. Eda. Lattice dynamics in mono-and few-layer sheets of WS_2 and WSe_2. *Nanoscale* **5**, 9677-9683 (2013).

20. F. Wang, I. A. Kinloch, D. Wolverson, R. Tenne, A. Zak, E. O. Connell, U. Bangert, and R. J. Young. Strain-induced phonon shifts in tungsten disulfide nanoplatelets and nanotubes. *2D Materials* **4**, 015007(2016).

21. X.Hu, Z.Hemmat,L.Majidi,J.Cavin,R.Mishra,A.S.Khojin,S.Ogut,andRobert F. Klie. Controlling nanoscale thermal expansion of monolayer transition metal dichalcogenides by alloy engineering. *Small* **16**, 1905892 (2020).

22. N. Zeng, Y. C. Wang, J. Neilson, S. M. Fairclough, Y. Zou, A. G. Thomas, R. J. Cernik, S. J. Haigh, and D. J. Lewis. Rapid and low-temperature molecular precursor approach toward ternary layered metal chalcogenides and oxides: $Mo_{1-x}WxS_2$ and $Mo_{1-x}W_xO_3$ Alloys ($0\leq x\leq 1$). *Chem. Mater.* **32**, 7895-7907(2020).

23. Y. Chen, D. O. Dumcenco, Y. Zhu, X. Zhang, N. Mao, Q. Feng, M. Zhang, et al. Composition-dependent Raman modes of $Mo_{1-x}W_xS_2$ monolayer alloys. Nanoscale6, 2833-2839 (2014).

24. X. Li, D. Jiang, J. Zhang, Q. Lin, Z. Chen, and Z. Huang. The dispersion of boron carbide powder in aqueous media. J. Eur. Ceram. Soc. 33, 1655-1663(2013).

25. V. Guerra, C. Wan, V. Degirmenci, J. Sloan, D. Presvytis, and T. McNally. 2D boron nitride nanosheets (BNNS) prepared by high-pressure homogenization: structure and morphology. *Nanoscale* 10, 19469-19477(2018).

26. Cui, X., Chen, G. and Han, X., Journal of Chemical & Engineering Data, 51, 1860-1861 (2006).

27. Nian, Q., Wang, Y., Yang, Y., Li, J., Zhang, M.Y., Shao, J., Tang, L. and Cheng, G.J., Scientific reports, 4, 1-8 (2014).

28. Santos, T., Costa, L.C., Valente, M., Monteiro, J., and Sousa, J., 3D electromagnetic field simulation in microwave ovens: a tool to control thermal runaway. In COMSOL Conference (2010).

29. Sun, J., Wang, W., and Yue, Q., Materials, 9, 231 (2016).

30. Lopez-Iturri, P., de Miguel-Bilbao, S., Aguirre, E., Azpilicueta, L., Falcone, F. and Ramos, V., BioMed research international, 2015.

31. Shahid, R., Gorlov, M., El-Sayed, R., Toprak, M.S., Sugunan, A., Kloo, L. and Muhammed, M., Materials Letters, 89, 316-319 (2012).

32. Asakuma, Y., Matsumura, S., Asada, M. and Phan, C., International Journal of Thermophysics, 39, 1-12(2018).

33. Fujimoto, Y. Advances in Condensed Matter Physics, 2015.

34. Yutomo, E.B., Noor, F.A. and Winata, T., RSC Advances, 11, 18371-18380 (2021).

35. Benti, N.E., Tiruye, G.A. and Mekonnen, Y.S., RSC Advances, 10, 21387-21398 (2020).

36. Yao, Y., Ao, K., Lv, P. and Wei, Q., Nanomaterials, 9, 844(2019).

37. Yu, Y., Nam, G.H., He, Q., Wu, X.J., Zhang, K., Yang, Z., Chen, J.,

Ma, Q., Zhao, M., Liu, Z. and Ran, F.R., Nature Chemistry, 10,638-643 (2018).
